# Exploring Coumarin-Based Boron Emissive Complexes as Temperature Thermometers in Polymer-Supported Materials

**DOI:** 10.3390/s23031689

**Published:** 2023-02-03

**Authors:** Gonçalo Pedro, Frederico Duarte, Dmitrii A. Cheptsov, Nikita Yu. Volodin, Ivan V. Ivanov, Hugo M. Santos, Jose Luis Capelo-Martinez, Cristián Cuerva, Elisabete Oliveira, Valerii F. Traven, Carlos Lodeiro

**Affiliations:** 1 BIOSCOPE Research Group, LAQV-REQUIMTE, Chemistry Department, NOVA School of Science and Technology, FCT NOVA, Universidade NOVA de Lisboa, 2829-516 Caparica, Portugal; 2 D. Mendeleev University of Chemical Technology of Russia, Miusskaya sq., 9125047 Moscow, Russia; 3 PROTEOMASS Scientific Society Caparica Campus, Rua dos Inventores, Madam Parque, 2829-516 Caparica, Portugal; 4 MatMoPol Research Group, Department of Inorganic Chemistry, Faculty of Chemical Sciences, Complutense University of Madrid, Ciudad Universitaria, 28040 Madrid, Spain

**Keywords:** coumarin derivatives, boron complexes, doped polymers, temperature sensors

## Abstract

Three coumarin-based boron complexes (L1, L2 and L3) were designed and successfully incorporated into polymeric matrixes for evaluation as temperature probes. The photophysical properties of the complexes were carried out in different solvents and in the solid state. In solution, compound L1 exhibited the highest fluorescence quantum yield, 33%, with a positive solvatochromism also being observed on the absorption and emission when the polarity of the solvent increased. Additionally in the presence of anions, L1 showed a colour change from yellow to pink, followed by a quenching in the emission intensity, which is due to deprotonation with the formation of a quinone base. Absorption and fluorescence spectra of L1 were calculated at different temperatures by the DFT/B3LYP method. The decrease in fluorescence of compound L1 with an increase in temperature seems to be due to the presence of pronounced torsional vibrations of the donor and acceptor fragments relative to the single bond with C(carbonyl)-C (styrene fragment). L1, L2 and L3, through their incorporation into the polymeric matrixes, became highly emissive by aggregation. These dye@doped polymers were evaluated as temperature sensors, showing an excellent fluorescent response and reversibility after 15 cycles of heating and cooling.

## 1. Introduction

The combination of conventional fluorophores into a single hybrid structure containing a difluoronated boron centre is a powerful approach to modulating the photophysical properties of such fluorophores [1]. In fact, the absorption and emission maximum wavelengths, solvatochromism and sensorial ability are photophysical properties that can be easily tuned through this combination.

Boron complexes have been widely explored for chemical sensing, drug discovery and optical materials and devices. Boron can coordinate with oxygen and nitrogen atoms through B-O and B-N bonds, respectively [2]. Bortezomib and Ixazomib for multiple myeloma treatment, Tavaborole for onychomycosis and Meropenem/Vaborbactam for bacterial infection treatments are examples of boron-containing drugs in the market [3].

Coumarin derivatives are biocompatible, with large Stokes shifts and high fluorescence quantum yields, but most of them are excitable in the UV-vis, region limiting their environmental and biological applications [4]. Thus, coumarin derivatives with excitation wavelengths from green to infrared have been designed by rigidifying with other fluorophores. Despite the enhanced properties, most dyes have a π-conjugated and rigid structure, leading to non-emissive compounds caused by aggregation. This is particularly evident in the solid state, where these molecules are in thin films [5]. Moreover, boron complexes of π-conjugated chelates are known as aggregation-induced emissive (AIE) active molecules [6].

Coumarin-based boron complexes with outstanding properties and applications have been reported in the literature. For example, a near-infrared fluorescent probe coumarin-based boron complex with positive solvatochromism and AIE effects, was reported by Lai et al. as a powerful tool for fluorescence monitorization of liquid droplets, imaging gastric fat in living obese mouse [7]. Tathe et al. published the synthesis of red-emitting dye derivatives of coumarin–boron complexes, where their properties were estimated by computational and solvatochromic assays [8]. Later, the same author explored the photochemical properties of the coumarin–carbazole chalcone dyes and their BF_2_ complexes in different solvents. Their results showed that the emission spectra were red-shifted as the polarity increased. When the donor group H was replaced by NEt_2,_ there was an evident red shift in both the absorption and emission spectra, which was attributed to efficient charge transfer (CT) as the electron-withdrawing power of the coumarin carbonyl group increased with the complexation of the BF_2_ group. Moreover, they proved that the emission intensity increases with higher viscosity [9]. These compounds showed promising properties for future applications in nonlinear optical materials, which can be used for nonlinear optical devices such as optical switches and modulators, because they showed sensitivity towards solvent polarity.

Despite the outstanding biological properties of metal complexes containing coumarin derivatives as core in the literature [10], the application of boron difluoride–coumarin complexes into thin polymeric films is practically inexistent.

Polymeric matrixes containing boron difluoride (BF_2_) adducts exhibit unique optical and electrochemical properties showing a wide range of applications, being applied in fluorescence imaging, spectroscopic sensing [11] and light harvesting [12].

Pyridyl-β-diketonate BF_2_ complexes doped in PMMA and SBS thin films were studied with temperature by Jiménez and coworkers [13]. As expected, the fluorescence decreased when temperature increased and recovered upon cooling. However, they found an interesting feature in the PMMA thin film, where an enhancement in the fluorescence was observed at high temperatures. This enhancement is due to AIE by the interactions between the dye and the polymer. Thermochromic properties were also witnessed in SBS at 165 °C with quenching in the emission at 478 nm, followed by the appearance of a new emissive peak at 423 nm. This behaviour can be explained by the SBS matrix properties. Since it shows elasticity, the boron difluoride compound can rearrange inside the polymer with increasing temperature, originating changes in luminescence. The results demonstrate great potential for future applications of these materials in temperature sensors [13].

In this work, the optical properties such as solvatochromism and anion-sensing ability of three coumarin-based boron complexes were evaluated. These complexes were further incorporated into polymeric matrixes and explored as temperature sensors. DFT calculations were also performed to understand the optical properties observed.

## 2. Materials and Methods

### 2.1. Chemicals and Starting Materials

Chloroform (Honeywell, Minneapolis, MN, USA, 99.0–99.4%, CAS 67-66-3), DMSO (Honeywell, 99.5%, CAS 67-68-5), Acetone (Honeywell, 99.5%, 67-64-1), Poly(methyl methacrylate) (PMMA) (Sigma-Aldrich, St, Louis, MO, USA, MW ~350,000, CAS 9011-14-7), Ethylenediamine tetraacetic acid (EDTA) (Alfa Aesar, Ward Hill, MA, USA, CAS 194491-31-1), Ethanol (Honeywell, 99.9%, CAS 64-17-5), tetrahydrofuran (THF) (PanReac, Barcelona, Spain, 99.0%, CAS 109-99-9), 1,4-Dioxane (Sigma-Aldrich, >99.0%, CAS 123-91-1), Dimethylformamide (DMF) (Sigma-Aldrich, 99.8%, CAS 68-12-2), LUDOX^®^ AS-30 colloidal silica (SiO_2_, Sigma-Aldrich, 30 wt.% suspension in water, CAS 7631-86-9), Acetonitrile (Merck Millipore, Darmstadt, Germany, 99.5%, CAS 75-05-8), Fluorescein (Fluka, Minneapolis, MN, USA, CAS 2190318), Rhodamine B (TCI Europe, Switzerland, CAS 201-383-9), Cresyl Violet (Exciton, Dayton, UK), Tetrabutylammonium cyanide (Sigma-Aldrich, CAS 10442-39-4), Tetrabutylammonium fluoride(Sigma Aldrich, CAS 22206-57-1), Tetrabutylammonium bromine (Sigma Aldrich, CAS 1643-19-2), Tetrabutylammonium chlorine (Sigma Aldrich, CAS 1112-67-0). 4-Hydroxybenzaldehyde (Sigma-Aldrich, 98.0%, CAS 123-08-0), Acetic acid (Sigma-Aldrich, glacial, ≥99.0%, CAS 64-19-7), Sulphuric acid (Sigma-Aldrich, ACS reagent, 95.0–98.0%, CAS 7664-93-9).

### 2.2. Instrumentation

The absorption spectra were recorded on a JASCO V-650 UV-Vis Spectrophotometer and the fluorescence emission spectra on a Horiba Jobin-Yvon Scientific Fluoromax-4. Spectra of solid samples were collected with a Horiba-Jobin-Yvon Fluoromax-4^®^ spectrofluorometer using an optic fibre connected to the equipment, by exciting the solid compounds at appropriated λ (nm). A correction for the absorbed light was performed when necessary. Lifetime studies were carried out on TemPro, Deltahub Nanoled of Horiba Jobin-Yvon, with a 455 nm Nanoled. All instruments were provided by Proteomass-BIOSCOPE facility.

The mass spectra were obtained using the Thermo Scientific mass spectrometer (ISQ LT Single Quadrupole Mass Spectrometer) by electron impact with ionizing electron energy of 70 eV.

The NMR spectra were recorded on a Bruker Avance spectrometer in DMSO-d_6_ at 25 °C using glass ampoules: ^1^H NMR spectrum at 500 MHz and ^13^C NMR spectrum at 126 MHz. An internal standard was used for tetramethylsilane (SiMe_4_) or a signal of residual protons and carbon of ^13^C solvent with respect to SiMe_4_ (for DMSO-d_6_: δ_H_ 2.50 ppm, δ_C_ 39.5 ppm). The following abbreviations were used to describe the spectrum ^1^H NMR: s—singlet, d—doublet, m—multiplet.

Melting points were measured on a Stuart melting point apparatus SMP30 and were uncorrected.

### 2.3. Synthetic Procedures

#### Synthesis of Compounds L1, L2 and L3

Synthesis of L1 (Figures S1–S4): To a suspension of boron difluoride complex, 3-acetyl-4-hydroxycoumarin (1260 mg, 5 mmol, 1 equiv.) in concentrated acetic acid (10 mL) and 4-hydroxybenzaldehyde (610 mg, 5 mmol, 1 equiv.) were added with stirring, and then concentrated H_2_SO_4_ (0.55 mL) was added dropwise. Next, the reaction mixture was heated at a boil for 5 h. The precipitate obtained was filtered off, washed with concentrated acetic acid (2 × 10 mL) and recrystallized in concentrated acetic acid. The substance was obtained in the form of a red powder, yield 1068 mg (60%), mp. 291–293 °C. HRMS (ESI), *m*/*z*: 357.0693 (M + H)^+^ (calcd for C_18_H_11_BF_2_O_5_H^+^: 357.0740). MS (ES+) calcd for C_18_H_11_BF_2_O_5_ *m*/*z*: 356.07, found *m*/*z*: 356.24. ^1^H NMR (DMSO-d_6_), δ (ppm): 6.90 (d, 2H, H(14,16), ^3^J = 8.8 Hz); 7.41–7.47 (m, 2H, H(6,8)); 7.68 (d, 2H, H(13,17), ^3^J = 8.8 Hz); 7.79–7.85 (m, 1H, H(7)); 8.01–8.20 (m, 3H, H(5,10,11)); 10.44 (br. signal, 1H, OH). ^13^C NMR (DMSO-d_6_), δ (ppm): 100.3; 116.3 (2C); 116.8; 117.9; 124.7; 125.4 (4C); 131.8 (2C); 134.2; 136.5; 138.3; 141.7; 148.0; 154.1.

Compounds L2 and L3, boron difluoride complex 3-acetyl-4-hydroxycoumarin have been completely characterized by NMR, IR, elemental analysis and mass spectrometry in [14,15,16]. The synthetic methodology has followed the same steps and sequential reactions.

### 2.4. Photophysical Characterization

The spectroscopic characterizations and titrations were performed using stock solutions of the compounds (ca. 10^−3^ M), prepared by dissolving the appropriate amounts of complexes L1, L2 and L3 in chloroform. The studied solutions were prepared by appropriate dilution of the stock solutions up to 10^−5^–10^−6^ M. L1 was also prepared in ethanol, dioxane, THF, DMF.

Titrations of complexes L1 to L3 were carried out in THF by the addition of microliter amounts of standard anion solutions of Br^−^, Cl^−^, F^−^ and CN^−^ in acetonitrile. All measurements were performed at 25 °C.

Fluorescence quantum yield of compounds L1, L2 and L3 were measured using a solution of Fluorescein [ϕ_F_ = 0.79], Rhodamine B [ϕ_F_ = 0.70] and cresyl violet [ϕ_F_ = 0.59] in ethanol as standards [17]. All solvents used were of the highest purity from Merck.

### 2.5. Kamlet–Taft Parameters

The solvent parameters (α, β, π*) used in the correlations are gathered in Table 1.

### 2.6. DFT Calculations

DFT and TD-DFT calculations were run with Gaussian 03 software package (Gaussian 03, Revision C.02, Inc., Wallingford, CT, USA, 2004) and Orca version 5.0.3 [18]. The molecular structure was optimized by using the B3LYP level [19,20] and the 6–31G** basis set [21] in vacuum. The solvent effect (toluene or Clhoroform) was also taken into account using CPCM [22] and the structure was optimized by DFT-B3LYP with the atom-pairwise dispersion correction with the Becke–Johnson damping scheme (D3BJ) [23,24]. The molecular electrostatic potential (MEP) surfaces, the natural bond orbital (NBO) atomic charges, the electronic distribution of the frontier orbitals, and the UV-Vis absorption and fluorescence spectra were determined from the optimized structures.

### 2.7. Preparation of Coumarin-Based Polymer Films

The coumarin-doped polymethylacrylate (PMMA) polymers were obtained at room temperature by dissolving 100 mg of PMMA in 5 mL of chloroform, followed by the addition of 1 mg of compounds L1, L2 or L3, previously dissolved in 1 mL of chloroform. The polymer films were obtained after slow evaporation at room temperature (~24 h). The procedure was repeated for KURARITY^TM^ LA4285 Kurashiki, Okayama, Japan, polymer for L1.

### 2.8. Temperature Variation Studies

The emission spectra of the doped polymer films with temperature variation were registered using a fibre optics probe connected to the spectrofluorometer and a heating plate with controlled temperature. A 1 × 1 cm square of each doped polymer film was cut and immobilized between two quartz glass slides and placed onto the heating plate for the measurements. The measures were performed from 25 °C to 180 °C and from 180 to 25 °C. Emission spectra were acquired at 10 °C intervals.

## 3. Results and Discussion

### 3.1. Photophysical Characterization

**Figure 1:**
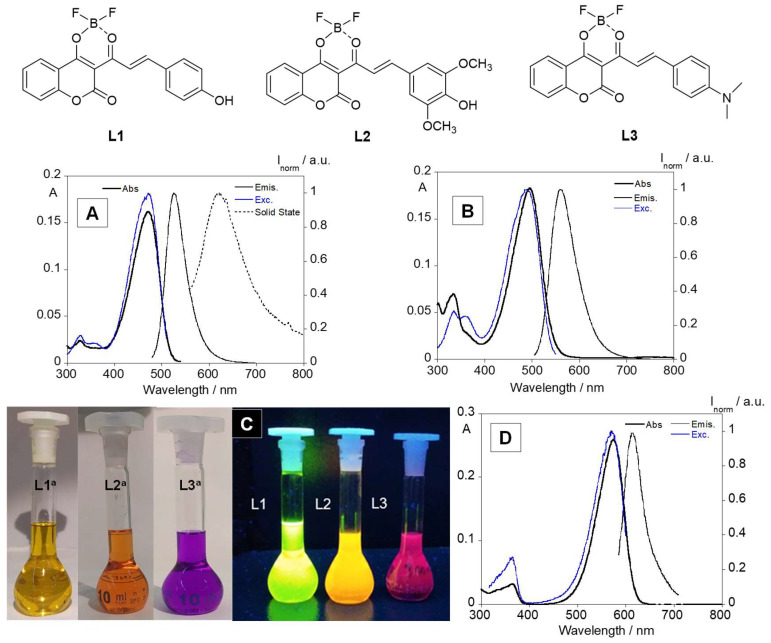
(**Top**) Chemical structure of coumarin-based boron complexes. Absorption, emission, excitation spectra and emission in chloroform (and solid state for L1) of complexes L1 (**A**), L2 (**B**) and L3 (**D**). (**C**) Image of the emission of three complexes under UV light (λ_exc_ = 365 nm). [L1] = [L2] = [L3] = 10^−5^ M; T = 25 °C.

The optical properties of coumarin-based boron complexes, L1, L2 and L3 were investigated in chloroform, and in THF, dioxane, ethanol, DMSO and DMF in the case of L1. As shown in Figure 1 and Table 2, complexes L1, L2 and L3 show the longest absorption band, centered at 470 nm (ε = 43,704 M^−1^.cm^−1^), 495 nm (ε = 30,266 M^−1^.cm^−1^) and 574 nm (ε = 93,735 M^−1^.cm^−1^), respectively, which is characteristic of the π conjugation skeleton produced by the coumarin-based boron complexes [15,25]. As can be seen in Figure 1C, the boron-chelated complexes showed an intense green, orange and red emission, with emission peaks at 526 nm, 560 nm and 613 nm for L1, L2 and L3, respectively. Additionally, from L1 to L3, it is possible to verify a red shift in the absorption and emission maximum peaks, which is attributed to the substituent-induced changes in the resonance system. The introduction of the inductive electron-donating groups (OH < OCH_3_ < N(CH_3_)_2_) lead to an increase in the electron density in the ground state, resulting in large dipole moment changes upon excitation and stronger solvatochromic effects [26].

Through the analysis of the absorption and excitation spectra for the three compounds, it is also possible to observe an overlap between the absorption spectra and the excitation spectra in the three cases, proving the absence of emissive impurities. Complex L1 was the only one to display emission in solid state with a single band at 624 nm, red-shifted in comparison to the emission spectra in solution. This red shift can be attributed to the close packing of the molecules in the solid-state analysis, as was observed previously in our group with other aromatic compounds [13,27].

The fluorescence quantum yields of the three compounds were also measured, using fluorescein, rhodamine B and cresyl violet as standards for complexes L1 to L3. The boron complexes L1, L2 and L3 exhibit a fluorescence quantum yield of 33%, 4% and 1%, respectively.

The fluorescence lifetime of L1, L2 and L3 were calculated (see Figure S5), and the values were of 0.32 ns, 0.11 ns and 0.03 ns, respectively.

The boron complex L1 was also characterized in other solvents (see Table 3). In all cases was observed a red shift (positive solvatochromism) on the absorption and emission spectra when the polarity of the solvent increased. Concerning the fluorescence quantum yield, L1 exhibited values of 33%, 32% and 22% in chloroform, THF and dioxane, respectively, decreasing as the solvent polarity decreased. These results indicate that solute and solvent interaction plays an important role since it stimulates significant changes in the position and intensity of absorption and emission bands [28]. In DMF, no emission was observed, which could be related to the higher dielectric constant (36.7) of the solvent, which is directly related to a polarity increase, leading to lower values of emission intensity.

Considering these results, the multiparametric fitting of the Kamlet–Taft equation (Equation (1)) was performed using the solvent parameters listed in Table 1. The Kamlet–Taft equation is one method used to quantitatively characterize solute–solvent interactions [29].

υ _=_ υ_0_ + a*α* + b*β* + pπ* (1)

where υ_0_ is the value of absorption and/or emission in a reference solvent, *α* is the hydrogen-bonding acceptor ability polarity scale; *β* is the hydrogen-bonding donor ability polarity scale; π* is the dipolarity/polarizability polarity scale; and the parameters a, b and p (corresponding to the responses of the solute property to the solvent property) are determined through a multiparametric fitting.

**Figure 2:**
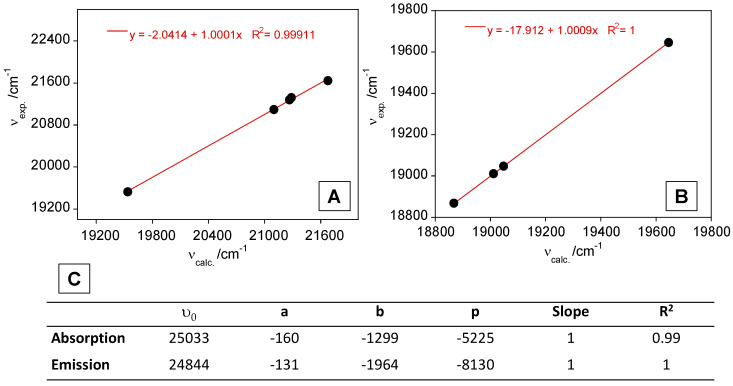
Plot of υ_exp_ versus υ_calc_ for absorption (**A**) and emission (**B**) data of L1. (**C**) υ_0_, a, b and p-values, in cm^−1^, slope and correlation coefficients obtained from Kamlet–Taft multiparametric fitting of the absorption and emission data.

Based on this fitting, linear plots of υ_exp_ versus υ_calc_ were obtained for L1 (Figure 2), and the fitted parameters (υ_0,_ a, b and p) and the slope and correlation coefficients are presented in Figure 2C.

As can be seen in Figure 2, the data fit the Kamlet–Taft model well, with R^2^ values of 0.99 and 1 for absorption and emission data.

### 3.2. Anion Sensing

**Figure 3:**
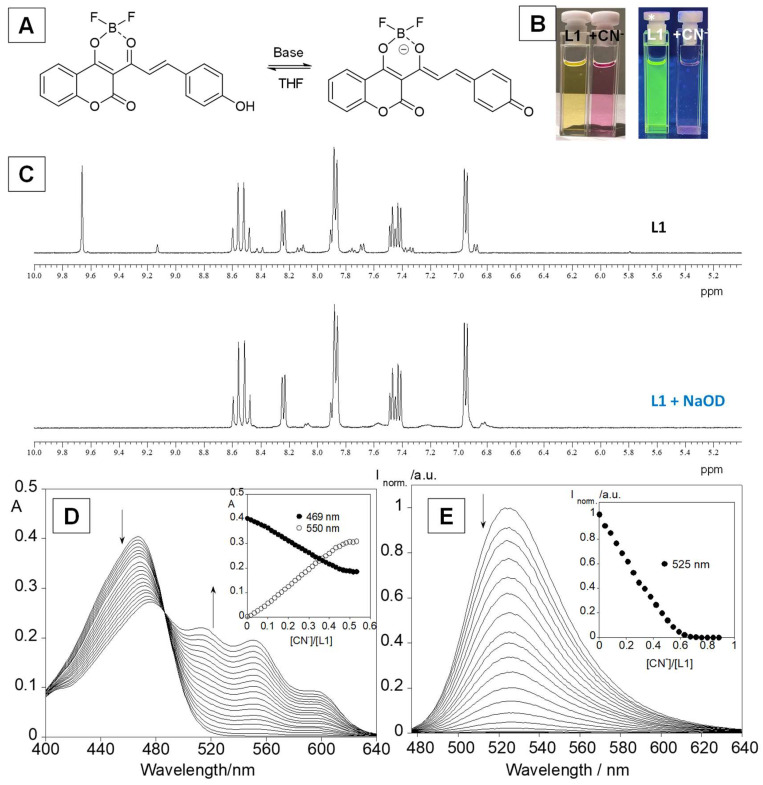
(**A**) Structures of the acid–base equilibrium of L1. (**B**) Naked-eye images and under a UV-light lamp of L1, L1 with the addition of CN^−^ anion. (**C**) NMR spectra of L1 and L1 + NaOD in THF. (**D**) spectrophotometric (**D**) and spectrofluorimetric (**E**) titrations of L1 with the addition of CN^−^ in THF. The inset (**D**,**E**) represents the absorption at 469 nm and 550 nm and the emission at 525 nm as function of [CN^−^]/[L1]. ([L1] = 1.0 × 10^−5^ M, λ_exc_= 469 nm, T = 25 °C).

Titrations of L1 with spherical (F^−^, Cl^−^, Br^−^), and linear (CN^−^) anions were carried out in THF, followed by the addition of small amounts of anion in acetonitrile. Figure 3 shows the spectral changes obtained in the ground and excited states obtained for L1 during the titration with CN^−^. In the absorption spectra, as the anions are added, a decrease is observed in the absorbance at 469 nm, followed by the appearance of a new band at ca. 550 nm. Concerning the excited state, a quenching in the emission intensity at ca. 525 nm is detected. These results are in accordance with colour changes, where a change of the original yellow to pink with a consequent disappearance of the green emission is visualized. This behaviour is due to the loss of protons that leads to the formation of a quinone base with increased conjugation that red shifts the absorption. This was confirmed by NMR titration by the disappearance of a peak at 9.64 ppm (see Figure 3A,E). Similar results were obtained for the other anions (see Figure S6).

### 3.3. Theoretical Calculations and Structural Analysis of L1

**Figure 4:**
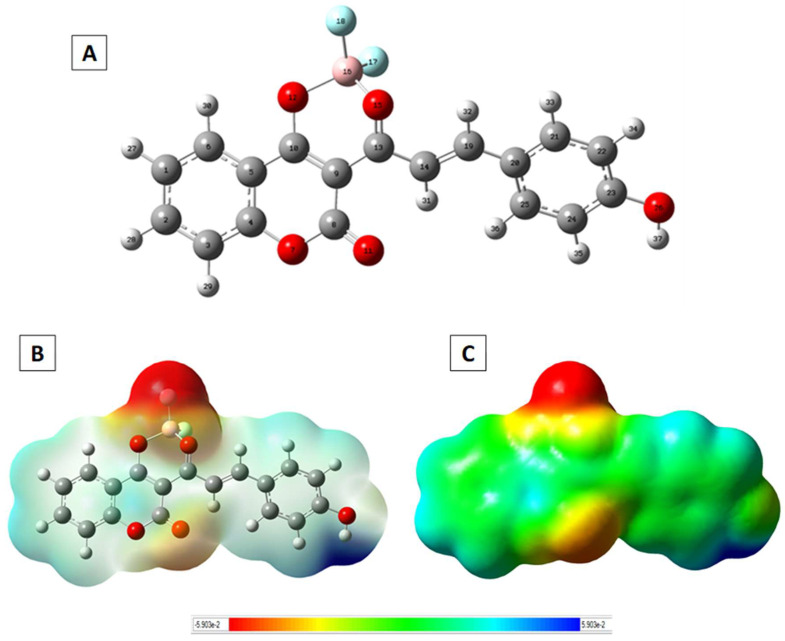
(**A**) Optimized molecular structure of L1, including the atom numbering; (**B**,**C**) molecular electrostatic potential maps obtained from the optimized structure.

The molecular structure and electronic properties of L1 were analysed by theoretical calculations using the DFT/B3LYP method with the 6–31G** basis set. As observed in Figure 4A, the β-diketonate ligand shows a great planarity; the coumarin moiety presents a small deviation with respect to the diketonate group (dihedral angle C8-C9-C13-O15 of 154.4°), and the benzene group is slightly rotated with respect to the diketone plane (torsion angle of 2.0°). The coordination to the BF_2_ fragment generates a six-membered cycle with B-F and B-O bond lengths of 1.5 Å, which agrees with diketonate-based borondifluoride complexes reported in the literature [30]. Selected bond lengths and angles are collected in the supporting information (Tables S1–S3).

The charge distribution in the structure of L1 is shown in Figure 4B,C by drawing the molecular electrostatic potential (MEP) surface, and Figure S7 collects the natural bond orbital (NBO) atomic charges. The highest negative charge region (dark red area) is mainly located at around the coordination environment, as expected due to the presence of the fluoride and oxygen atoms, which have high negative charge values of −0.66 e and −0.55 e, respectively. The carbon atoms of the benzene groups present charge values ranging between −0.1 and −0.3 e, which is consistent with a π-delocalized system. Note that the area over the atoms O7, C8 and O11 from the coumarin group also shows a certain negative charge density, which could favour the establishment of intermolecular interactions between the benzene groups of neighbouring molecules. On the other hand, the most positive region (dark blue area) appears over the atom H37 (atomic charge value of 0.5 e), making this proton susceptible to nucleophilic attacks.

Figure S8 displays the electronic distribution in the HOMO and LUMO frontier orbitals, which are highly localized over the atoms C8 and O11 from the coumarin group and the atoms C9, C13, C14 and O15 from the diketonate moiety, respectively. TD-DFT studies reveal that the S_0_ → S_1_ transition is mainly dominated by a HOMO → LUMO excitation, with an energy vertical excitation value of 2.97 eV (λ = 417.92 nm) and a singlet oscillator strength value of 0.9204. The remaining nonzero oscillator strength excitation corresponds to S_0_ → S_2_ and S_0_ → S_3_ transitions at 3.34 eV (λ = 370.70 nm) and 3.44 eV (λ = 360.45 nm), respectively (Table S4).

**Figure 5:**
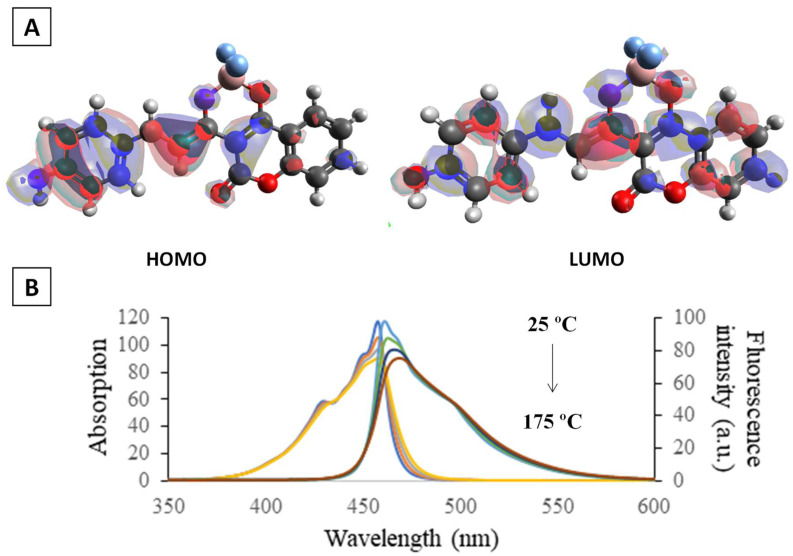
(**A**) Molecular orbital plots of the HOMO and LUMO of L1; (**B**) the absorption and fluorescence spectra of the compound L1 calculated at different temperatures.

The structure of L1 was also optimized by using the DFT/B3LYP method with the 6–31G** basis set and the correction for the pairwise dispersion of atoms with the Becke–Johnson damping scheme (D3BJ) [23,24], considering the effect of the solvent (toluene or chloroform) within the framework of CPCM. As observed in Figure 5A, the electron density in the ground state is localized on the substituent at position 3 of coumarin. In the excited state, the electron density is distributed throughout the molecule. The absorption and fluorescence spectra were calculated at different temperatures in increments of 50 °C (Figure 5B). The calculation results show a systematic decrease in the intensity of both absorption and fluorescence with an increase in temperature.

To explain the reason for the decrease in fluorescence of compound L1 with an increase in temperature, we turn to the analysis of IR spectra. The results of quantum chemical calculations show the presence of pronounced torsional vibrations of the donor and acceptor fragments relative to the single bond with C(carbonyl)-C (styrene fragment), which are characterized by a band of 29 cm^−1^ in the IR spectrum. The range of fluctuations at normal temperature reaches 25 °C. One can assume that with heating, this range can increase up to complete loss of conjugation of these fragments. It leads to the loss of emission when the temperature increases. The mentioned fluctuations are also well seen in the dimer (see Figure S9), and can be proposed in the PMMA film as well. The calculation results fully correspond to the observed spectral changes.

### 3.4. Solid Supports and Temperature Studies

**Figure 6:**
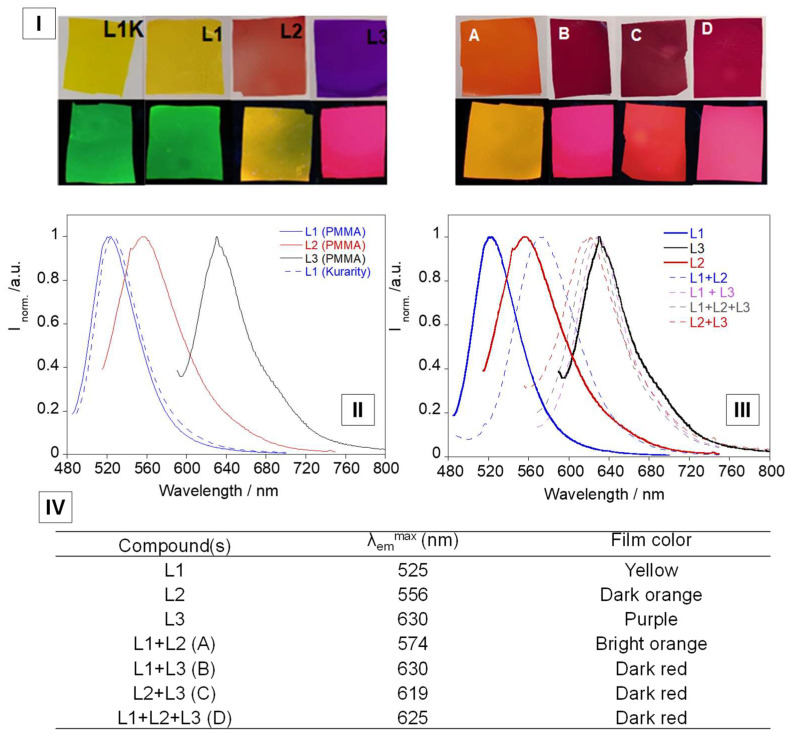
(**I**) Picture of compounds L1, L2 and L3 and mixtures in PMMA polymer films. L1K represents L1 in KURARITY^TM^ polymer (above: naked-eye, below: under UV lamp). (**II**) Emission spectra of L1, L2, L3 doped in PMMA and L1 doped in Kurarity polymer films. (**III**) Emission spectra of the mixtures doped in PMMA polymer films. (**IV**) Emission peak wavelengths and colours of the different doped PMMA polymers. Mixtures: (A) L1+L2, (B) L1+L3 (C) L2+L3 (D) L1+L2+L3.

PMMA polymers were doped with coumarin–boron complexes L1, L2 and L3, as well as with mixtures of all combinations between the three complexes, and then characterized by solid-state emission. KURARITY^TM^ LA4285 polymer was also doped with compound L1 and characterized (see Figure 6I,II). The results with this polymer are similar to those obtained with the PMMA polymer. The emission spectra in both display a very similar band, with a single peak at 525 nm for PMMA polymer and at 527 nm for the KURARITY^TM^ polymer.

As presented in Figure 6, complexes L1 and L2 showed similar emission peaks to those observed in chloroform solution. On the other hand, in the case of L3, the doped PMMA polymer presented a ~15 nm red shift.

In the case of the polymer films that were doped with different combinations of the compounds, all of the emission spectra of the mixtures (Figure 6) experienced a red shift, when compared to each of their individual emission spectra, as can be seen in Figure 6IV, with the exception of the mixtures with compound L3.

Taking into account the good results obtained in solution and in order to increase the analytical applications of these complexes, the thermal sensing capacity of the PMMA-based doped films was evaluated. The emission spectra were performed from 26 °C to 180 °C and from 170 °C to 30 °C to evaluate the reversibility of the temperature sensing.

**Figure 7:**
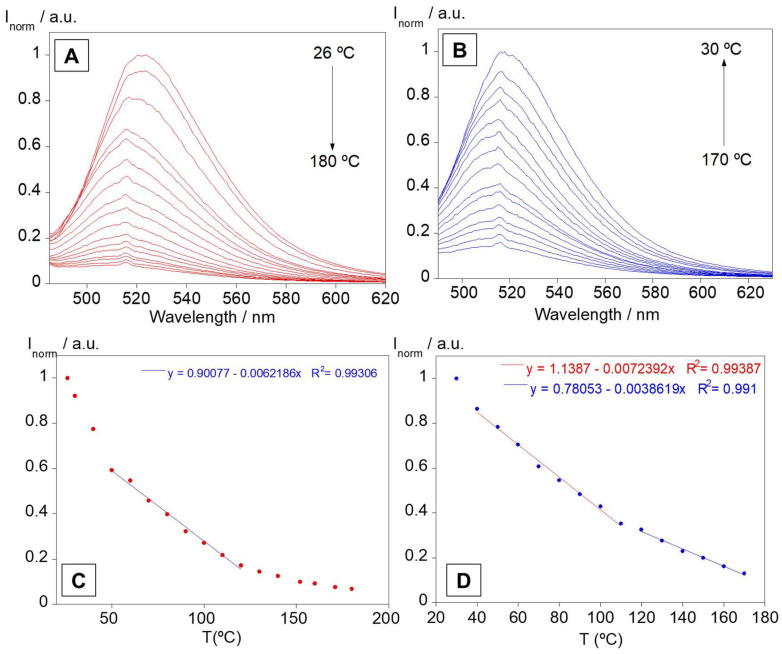
Emission spectra of PMMA@L1 during heating (**A**) and cooling (**B**). Emission intensities at 525 nm, with calibration curves, as a function of temperature for heating (**C**) and cooling (**D**).

Figure 7 displays the spectra for heating (Figure 7A) and cooling (Figure 7B), as well as the emission intensity at 525 nm as a function of temperature (Figure 7C,D), for the PMMA@L1 polymer. Temperature increasing lead to quenching in the emission intensity. Moreover, in the cooling phase, PMMA@L1 polymer showed a recovery of the emission intensity of ca. 83%. A linear relation between emission intensity and temperature was visualized from 60 °C to 120 °C in heating and in the range of 40–110 °C and 120–180 °C in cooling, evidencing the capacity of this polymer film to be used as a temperature sensor in the solid state. This fluorescent responsive behaviour also exhibited excellent reversibility after 15 cycles of heating and cooling (see Figure S10).

The other polymer films (see Figures S11–S17) also demonstrated similar behaviour to that of compound L1. The recovery of emission intensity was close to 100% in the polymer films (except for mixture L1+L2), and superior to 100% in some cases, which indicates that these films have the capability to maintain their emissive properties after a heating and cooling cycle (see Table S5).

Most of these films expressed an increase in emission intensity within the first 30–50 °C of heating, which can be attributed to a rearrangement of the compounds into the polymer leading to more planar structures. Overall, all doped PMMA polymers had their optical properties fully recovered after the temperature measurement cycle, with a linear behaviour between emission intensity and temperature in various regions, opening a range of applications as low-cost temperature indicators for smart labels.

## 4. Conclusions

To sum up, three coumarin-based boron complexes were successfully evaluated in solution and in the solid state. In the presence of anions, all complexes showed a colourimetric behaviour, with a change in colour from yellow to pink and a quenching in the emission intensity. Moreover, in solution, L1 was the only complex showing a significant fluorescence signal. Based on these results, L1 was evaluated in different solvents, revealing a positive solvatochromism. Incorporation of the complexes in polymeric matrixes led to emissive polymers by aggregation-induced emission (AIE), even in the case of L2 and L3, where in solution, no emission was observed. This is a very promising result, since most dyes have π-conjugated systems leading to non-emission compounds when they are in thin films. The behaviour of L1 was also evaluated by DFT, and the absorption and fluorescence spectra were calculated at different temperatures.

In general, when heating, the whole molecular energy increased, and the molecules had a high degree of freedom to tune the excited-state conformation. In this process, the excited-state energy was consumed, leading to weaker emission. Particularly, the decrease in fluorescence of compound L1 with the increase in temperature seems to be due to the presence of pronounced torsional vibrations of the donor and acceptor fragments relative to the single bond with C(carbonyl)-C (styrene fragment). With heating, these vibrations are increased up to an expressed loss of conjugation of donor and acceptor fragments.

Based on the aforementioned results, all complexes@polymers were submitted to several cycles of heating and cooling stages, showing in all cases a total recovery of the emission and reversibility, opening a range of applications as low-cost temperature probes.

## Figures and Tables

**Table 1:** Solvent parameters (α: the solvent’s HBD acidity; β: the solvent’s HBA basicity; π*: the solvent’s dipolarity/polarizability; ε_r_: dielectric constant; η: refractive index).

Solvent	α	β	π*	ε_r_	η
**DMF**	0	0.69	0.88	36.7	1.4305
**Ethanol**	0.86	0.75	0.54	24.3	1.3610
**Chloroform**	0.2	0.1	0.69	9.08	1.4459
**THF**	0	0.55	0.58	7.52	1.4072
**Dioxane**	0	0.37	0.55	2.25	1.4224

**Table 2:** Photophysical properties of boron complexes L1, L2 and L3 in chloroform.

	λ_abs_^max^ (nm)	λ_em_^max^ (nm)	Δλ (nm)	Stokes Shift (cm^−1^)	λ_exc_^max^ (nm)	Φ_f_ (%)	τ_f_ (ns)
**L1**	470	526	56	178571	472	33	0.32
**L2**	495	560	65	153846	492	4	0.11
**L3**	574	613	39	256410	572	1	0.03

**Table 3:** Photophysical properties of boron complexes L1, L2 and L3 in the different solvents.

L1	λ_abs_^max^ (nm)	λ_em_^max^ (nm)	Δλ (nm)	λ_exc_^max^ (nm)	Color	Φ_f_ (%)	τ_f_ (ns)
Chloroform	470	526	56	472	Yellow	33	0.32
THF	469	525	56	471	Yellow	32	0.45
Dioxane	462	509	47	464	Yellow	22	0.34
Ethanol	474	530	56	472	Yellow	15	0.37
DMF	512/548	No emission			Orange	No emission	

## Data Availability

The data presented in this study are available online within this article or in the Supplementary Materials.

## Supplementary Materials

The following supporting information can be downloaded at: https://www.mdpi.com/article/10.3390/s23031689/s1, Figure S1. Mass spectrum of L1; Figure S2. ^1^H NMR spectrum of L1 in DMSO-d^6^; Figure S3. ^13^C NMR spectrum of L1 in DMSO-d_6_; Figure S4. HSQC spectrum of L1 in DMSO-d_6;_ Figure S5. Fluorescence lifetime decays and fitting obtained at 25 °C for L1, L2 and L3 in chloroform; Figure S6. Spectrophotometric (A, C, E) and spectrofluorimetric (B, D, F) titrations of L1 with the addition of Br^−^ (A, B), Cl^−^ (C, D) and F^−^ (E, F) in THF. The inset (B, D and F) represents the emission at 525 nm, as function of [Br^−^]/[L1], [Cl^−^]/[L1] or [F^−^]/[L1]. ([L1] = 1.0 × 10^−5^ M, λ_exc_ = 469 nm, T = 25 °C.); Table S1. Selected bond lengths for L1; Table S2. Selected bond angles for L1; Table S3. Selected dihedral angles for L1; Table S4. Calculated TD-DFT singlet vertical excitation wavelengths and corresponding singlet oscillator strengths for complex L1; Figure S7. Calculated NBO atomic charges for L1; Figure S8. Molecular orbital surfaces for the main frontier orbitals (HOMO and LUMO) involved in the S_0_–S_n_ transitions with the highest singlet oscillator strength; Figure S9. Orientation of the L1 molecules in the dimer; Figure S10. Fluorescence quenching and enhancement cycles with temperature of the L1@PMMA polymer (λ_em_  =  510 nm); Figure S11. Temperature-dependent emission spectra of L1 in KURARITY^TM^ 4285 (A) during heating and (B) during cooling, with calibration curves for heating (A1) and cooling (B1) at 525 nm; Figure S12. Temperature-dependent emission spectra of L2 in PMMA (A) during heating and (B) during cooling, with calibration curves for heating (A1) and cooling (B1) at 569 nm; Figure S13. Temperature-dependent emission spectra of L3 in PMMA (A) during heating and (B) during cooling, with calibration curves for heating (A1) and cooling (B1) at 640 nm; Figure S14. Temperature-dependent emission spectra of L1+L2 in PMMA (A) during heating and (B) during cooling, with calibration curves for heating (A1) and cooling (B1) at 552 nm; Figure S15. Temperature-dependent emission spectra of L1+L3 in PMMA (A) during heating and (B) during cooling, with calibration curves for heating (A1) and cooling (B1) at 607 nm; Figure S16. Temperature-dependent emission spectra of L2+L3 in PMMA (A) during heating and (B) during cooling, with calibration curves for heating (A1) and cooling (B1) at 628 nm; Figure S17. Temperature-dependent emission spectra of L1+L2+L3 in PMMA (A) during heating and (B) during cooling, with calibration curves for heating (A1) and cooling (B1) at 627 nm; Table S5. Interval(s) of temperature with linear behaviour between T °C and emission intensity of doped PMMA polymer films and percentage of emission recovery after cooling. Figure S18. The blue colour indicates the bonds with respect to which the dihedral angle changed; Figure S19. The changes in the energy difference between the HOMO and the LUMO (left) and the position of the absorption maximum (right) with a change in the dihedral angle relative to the bond; Figure S20. The changes in the energy difference between the HOMO and the LUMO (left) and the position of the absorption maximum (right) with a change in the dihedral angle relative to the bond B.
