# Supplementary material for: Exploring Coumarin-Based Boron Emissive Complexes as Temperature Thermometers in Polymer-Supported Materials
Source: Sensors (Basel). 2023 Feb 3;23(3):1689. doi: 10.3390/s23031689 (PMC9921380; doi:10.3390/s23031689)
Supplement: Supplementary file 1 [file sensors-23-01689-s001.zip › sensors-2133066-supplementary.pdf]

Supplementary Materials

Exploring Coumarin-Based Boron Emissive Complexes as Temperature Thermometers in Polymer-Supported Materials

Gonçalo Pedro [1], Frederico Duarte [1], Dmitrii A. Cheptsov [2], Nikita Yu. Volodin [2], Ivan V. Ivanov [2], Hugo M. Santos [1,3], Jose Luis Capelo-Martinez [1,3], Cristián Cuerva [4], Elisabete Oliveira [1,3], Valerii F. Traven [2,*] and Carlos Lodeiro [1,3,*]

[1] BIOSCOPE Research Group, LAQV-REQUIMTE, Chemistry Department, NOVA School of Science and Technology, FCT NOVA, Universidade NOVA de Lisboa, 2829-516 Caparica, Portugal
[2] D. Mendeleev University of Chemical Technology of Russia, Miusskaya sq., 9125047 Moscow, Russia
[3] PROTEOMASS Scientific Society Caparica Campus, Rua dos Inventores, Madam Parque, 2829-516 Caparica, Portugal
[4] MatMoPol Research Group, Department of Inorganic Chemistry, Faculty of Chemical Sciences, Complutense University of Madrid, Ciudad Universitaria, 28040 Madrid, Spain

* Correspondence: valerii.traven@gmail.com (V.F.T.); cle@fct.unl.pt (C.L.)

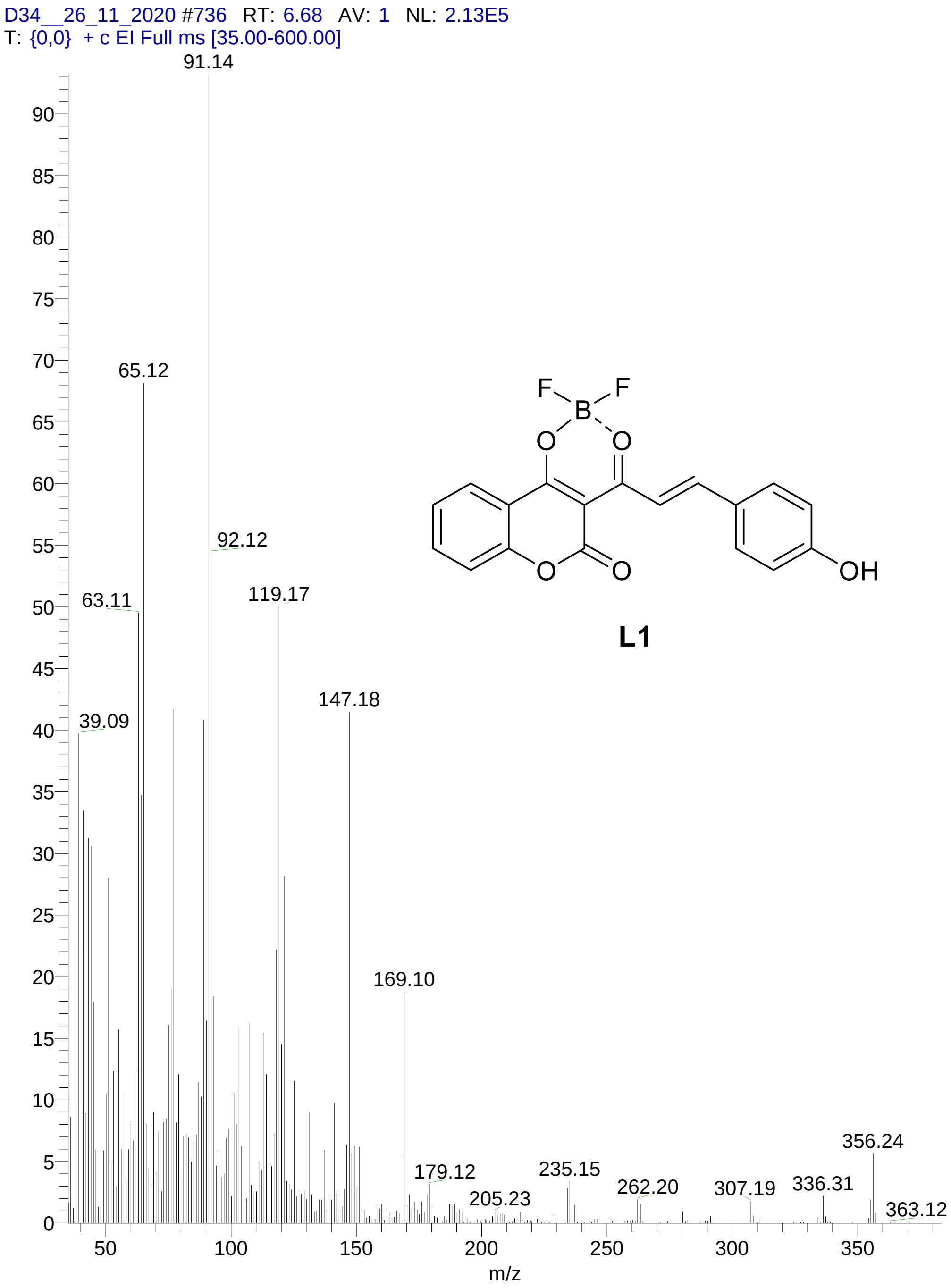

Figure S1. Mass spectrum of **L1.**

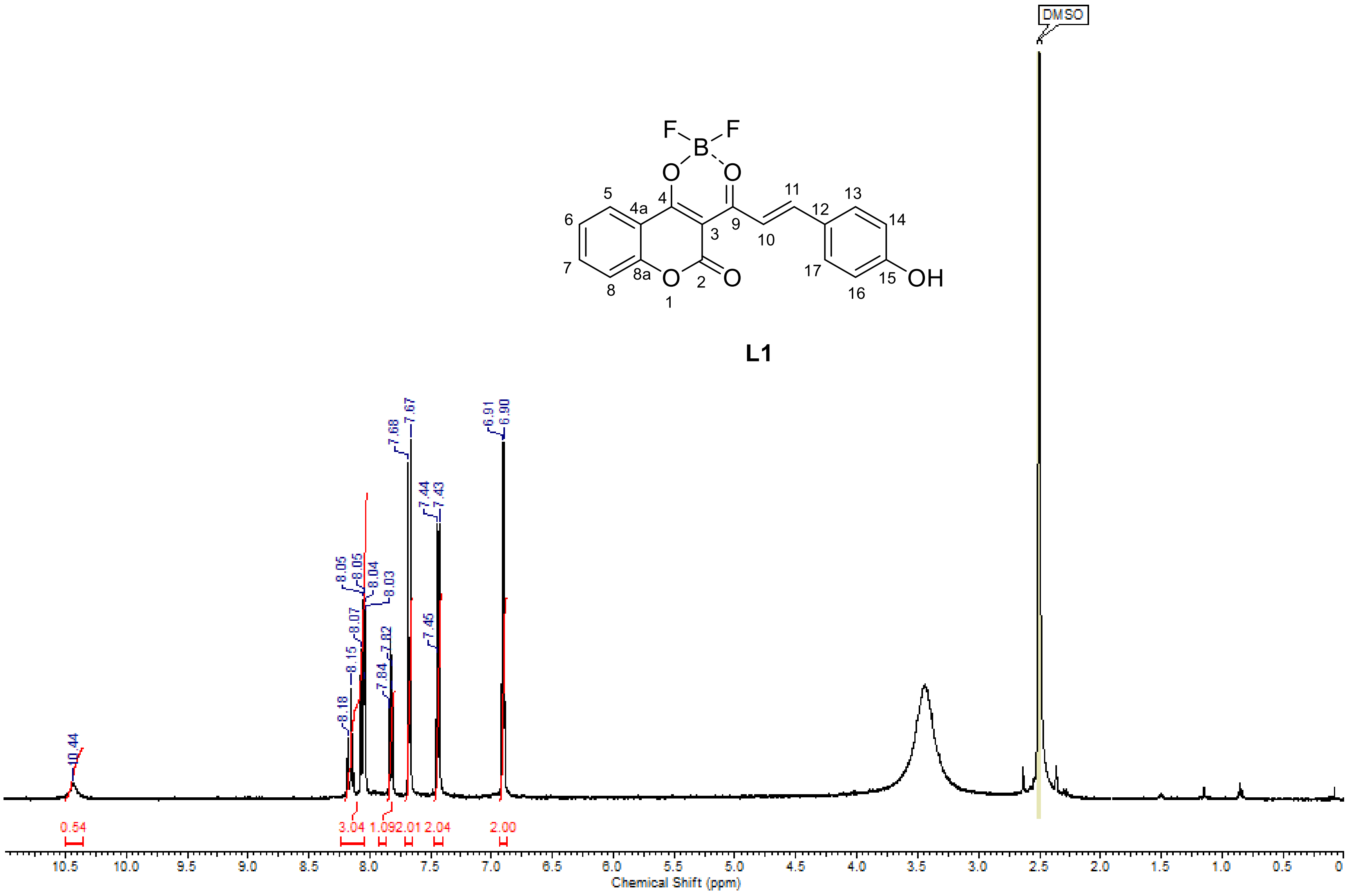

Figure S2. ^{1}H NMR spectrum of **L1** in DMSO-d_6.

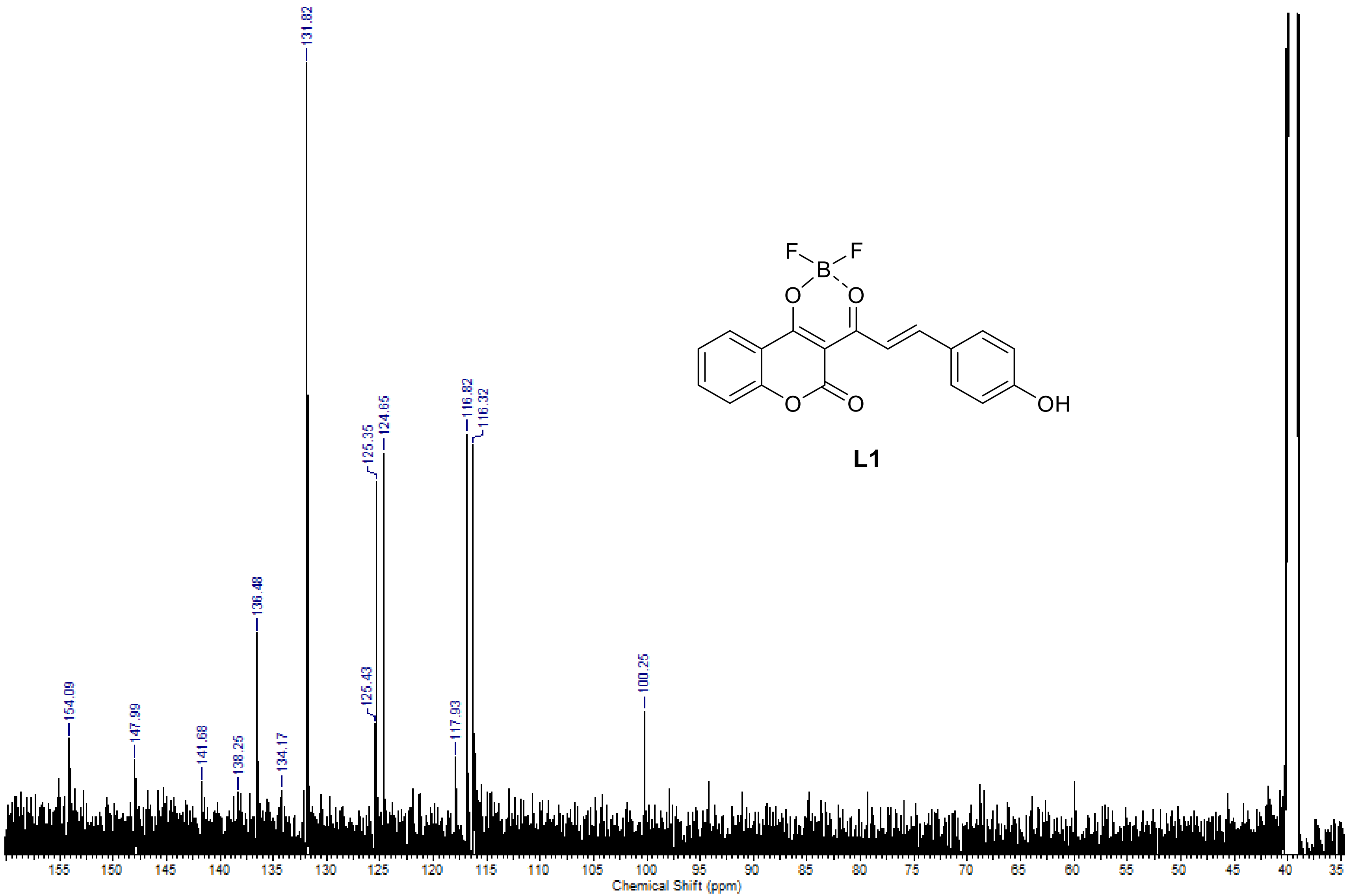

Figure S3. ^{13}C NMR spectrum of **L1** in DMSO-d_6.

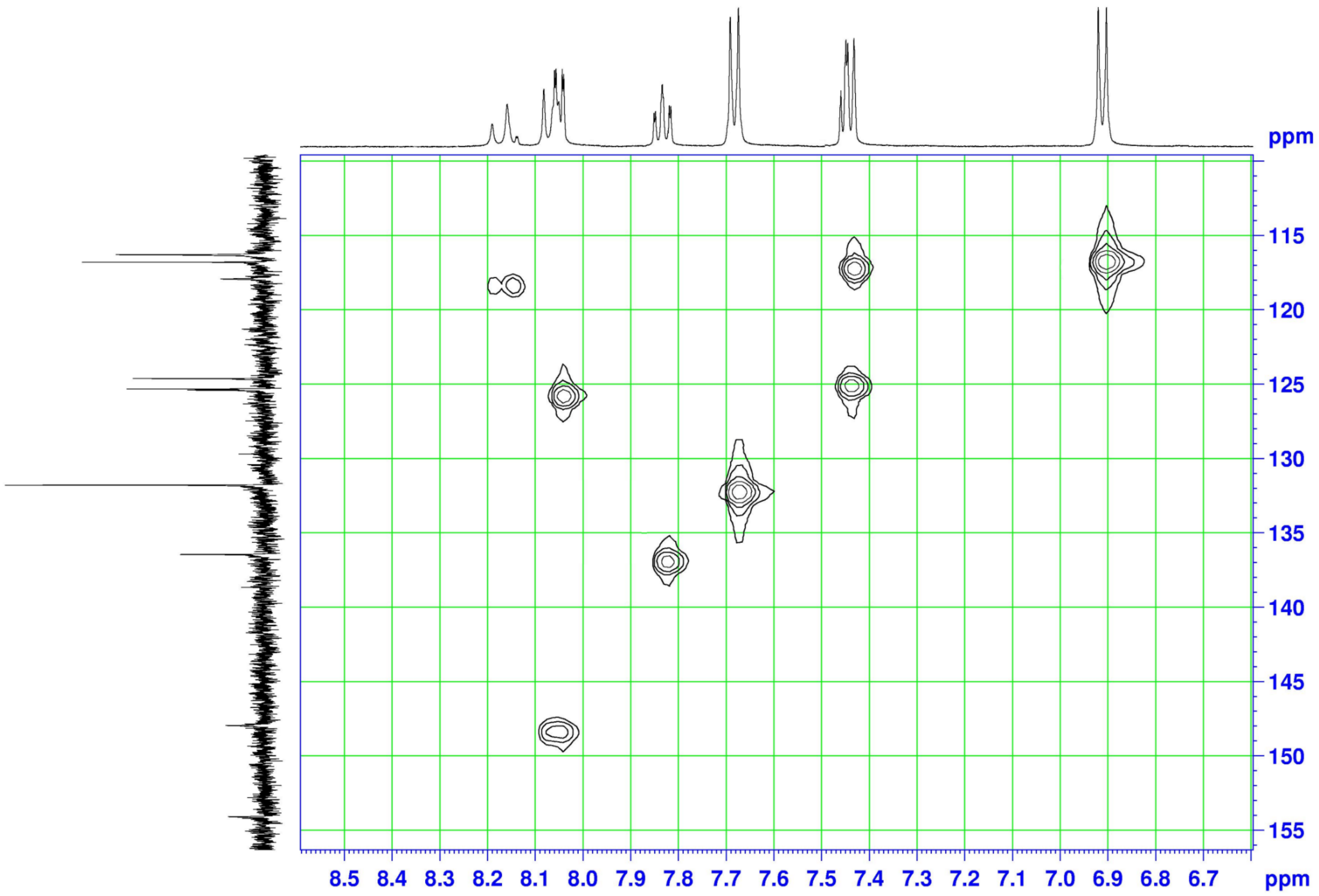

Figure S4. HSQC spectrum of **L1** in DMSO-d6.

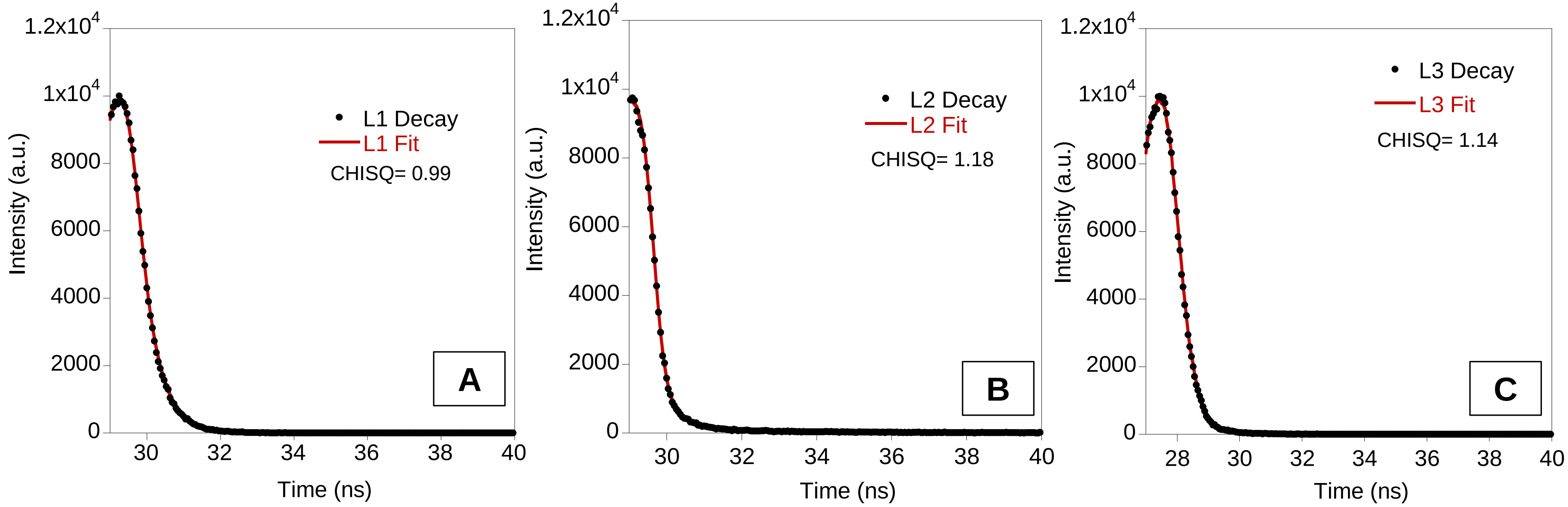

Figure S5. Fluorescence Lifetime decays~~, and~~ fitting <u>and CHISQ</u> obtained at 25ºC for L1, L2 and L3 in chloroform.

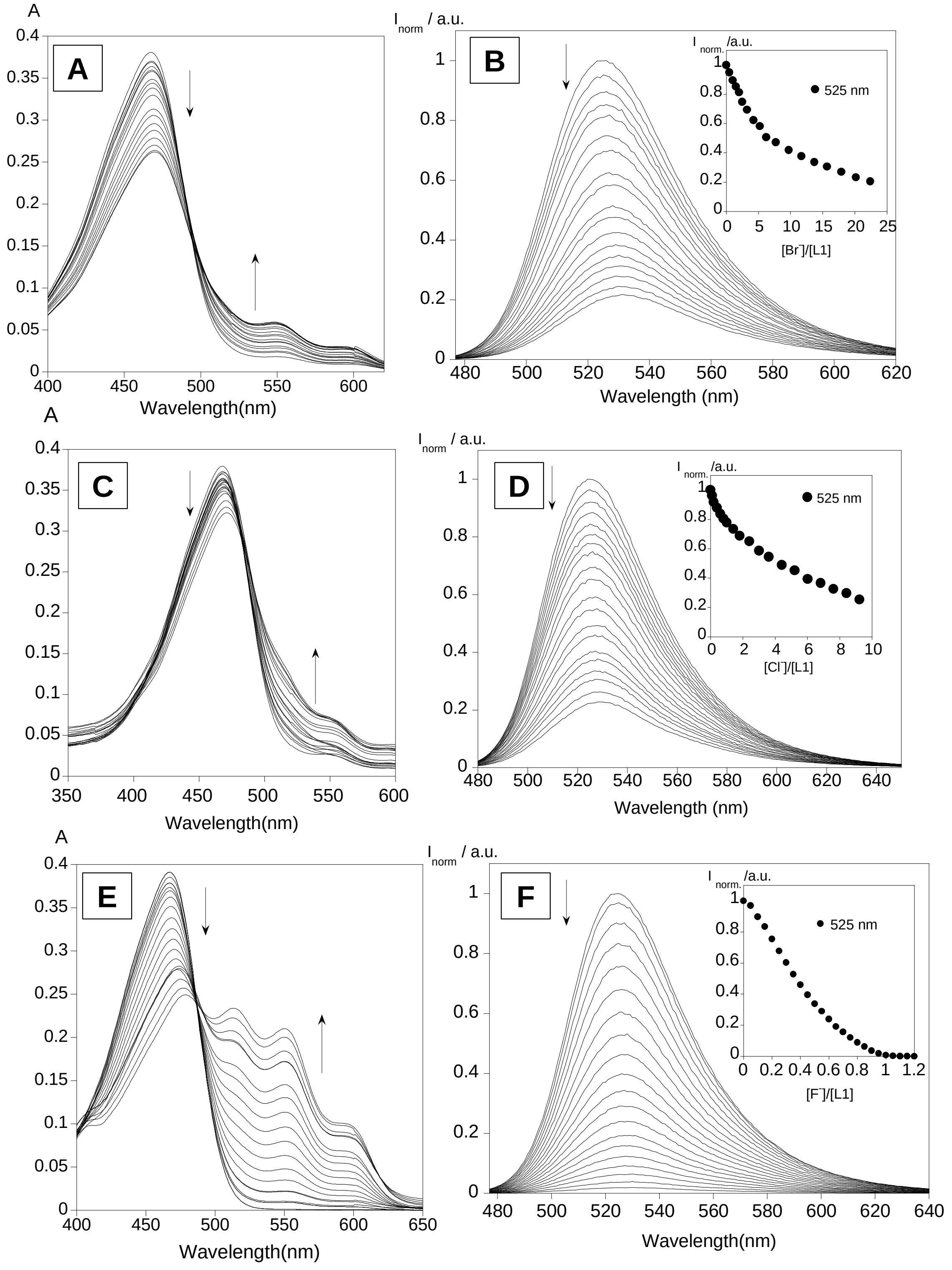

Figure S6. Spectrophotometric (A, C, E) and spectrofluorimetric (B, D, F) titrations of L1 with the addition of Br^- (A, B), Cl^- (C, D) and F^- (E, F) in THF. The inset (B, D and F) represents the emission at 525 nm, as function of [Br^-]/[L1], [Cl^-]/[L1] or [F^-]/[L1]. ([L1] = 1.0 ×10^{-5} M, λ_{exc}= 469 nm, T = 25ºC).

Table S1. Selected bond lengths for L1.

Atoms	d / A	Atoms	d / A
C(25)-H(36)	1.1007	C(9)-C(13)	1.3789
C(24)-H(35)	1.1036	C(10)-O(12)	1.3598
C(22)-H(34)	1.1033	O(7)-C(4)	1.3570
C(21)-H(33)	1.1043	C(8)-O(7)	1.3659
C(20)-C(25)	1.3478	C(9)-C(8)	1.3730
C(24)-C(25)	1.3427	C(8)-O(11)	1.2174
C(23)-C(24)	1.3422	C(10)-C(9)	1.3525
C(22)-C(23)	1.3421	C(5)-C(10)	1.3522
C(21)-C(22)	1.3420	C(5)-C(4)	1.3455
C(20)-C(21)	1.3494	O(26)-H(37)	0.9709
C(19)-C(20)	1.3522	C(23)-O(26)	1.3600
C(19)-H(32)	1.1039	C(6)-H(30)	1.1030
C(14)-H(31)	1.0962	C(3)-H(29)	1.1033
C(14)-C(19)	1.3497	C(2)-H(28)	1.1026
C(13)-C(14)	1.3728	C(1)-H(27)	1.1030
B(16)-F(18)	1.5448	C(1)-C(6)	1.3415
B(16)-F(17)	1.5450	C(5)-C(6)	1.3488
B(16)-O(15)	1.4726	C(3)-C(4)	1.3462
B(16)-O(12)	1.6382	C(2)-C(3)	1.3398
C(13)-O(15)	1.2377	C(1)-C(2)	1.3390

Table S2. Selected bond angles for L1.

Atoms	Angle / º	Atoms	Angle / º
H(37)-O(26)-C(23)	109.0409	C(14)-C(13)-O(15)	116.5479
H(35)-C(24)-C(25)	119.4918	C(14)-C(13)-C(9)	118.8289
H(35)-C(24)-C(23)	118.8453	O(15)-C(13)-C(9)	124.2294
C(25)-C(24)-C(23)	121.6629	B(16)-O(12)-C(10)	114.3226
C(24)-C(23)-C(22)	117.0202	C(13)-C(9)-C(8)	123.9286
C(24)-C(23)-O(26)	121.1728	C(13)-C(9)-C(10)	119.0990
C(22)-C(23)-O(26)	121.8068	C(8)-C(9)-C(10)	116.8019
H(34)-C(22)-C(23)	119.0047	O(7)-C(8)-C(9)	123.0980
H(34)-C(22)-C(21)	119.6320	O(7)-C(8)-O(11)	115.4104
C(23)-C(22)-C(21)	121.3633	C(9)-C(8)-O(11)	121.3893
H(36)-C(25)-C(20)	122.3985	O(12)-C(10)-C(9)	120.7339
H(36)-C(25)-C(24)	115.8792	O(12)-C(10)-C(5)	117.8909
C(20)-C(25)-C(24)	121.7221	C(9)-C(10)-C(5)	121.3665
H(33)-C(21)-C(22)	116.8929	H(30)-C(6)-C(1)	116.6143
H(33)-C(21)-C(20)	121.0935	H(30)-C(6)-C(5)	121.7500
C(22)-C(21)-C(20)	122.0137	C(1)-C(6)-C(5)	121.6356
C(25)-C(20)-C(21)	116.2177	C(4)-O(7)-C(8)	116.7402
C(25)-C(20)-C(19)	124.9044	C(10)-C(5)-C(4)	120.1155
C(21)-C(20)-C(19)	118.8777	C(10)-C(5)-C(6)	121.1358
C(20)-C(19)-H(32)	115.2030	C(4)-C(5)-C(6)	118.7475
C(20)-C(19)-C(14)	127.7488	O(7)-C(4)-C(5)	121.4902
H(32)-C(19)-C(14)	117.0456	O(7)-C(4)-C(3)	119.3276
F(18)-B(16)-F(17)	108.7894	C(5)-C(4)-C(3)	119.1804
F(18)-B(16)-O(15)	109.4290	H(29)-C(3)-C(4)	119.1303
F(18)-B(16)-O(12)	109.4496	H(29)-C(3)-C(2)	119.0091
F(17)-B(16)-O(15)	110.7266	C(4)-C(3)-C(2)	121.8606
F(17)-B(16)-O(12)	110.3274	H(28)-C(2)-C(3)	120.5432
O(15)-B(16)-O(12)	108.1018	H(28)-C(2)-C(1)	120.4487
B(16)-O(15)-C(13)	110.1149	C(3)-C(2)-C(1)	119.0080
H(31)-C(14)-C(19)	115.8253	H(27)-C(1)-C(6)	120.4386
H(31)-C(14)-C(13)	118.7681	H(27)-C(1)-C(2)	119.9940
C(19)-C(14)-C(13)	125.3374	C(6)-C(1)-C(2)	119.5674

Table S3. Selected dihedral angles for L1.

Atoms	Angle / º	Atoms	Angle / º
C(19)-C(20)-C(25)-C(24)	-179.8735	H(32)-C(19)-C(20)-C(21)	-0.6357
C(19)-C(20)-C(25)-H(36)	-0.0190	H(32)-C(19)-C(20)-C(25)	179.1787
C(21)-C(20)-C(25)-C(24)	-0.0546	C(13)-C(14)-C(19)-H(32)	1.8173
C(21)-C(20)-C(25)-H(36)	179.8000	C(13)-C(14)-C(19)-C(20)	-178.8037
C(23)-C(24)-C(25)-C(20)	-0.0536	H(31)-C(14)-C(19)-H(32)	178.7388
C(23)-C(24)-C(25)-H(36)	-179.9171	H(31)-C(14)-C(19)-C(20)	-1.8822
H(35)-C(24)-C(25)-C(20)	179.9282	C(9)-C(13)-C(14)-C(19)	-178.2026
H(35)-C(24)-C(25)-H(36)	0.0647	C(9)-C(13)-C(14)-H(31)	4.9587
O(26)-C(23)-C(24)-C(25)	179.9506	O(15)-C(13)-C(14)-C(19)	8.6931
O(26)-C(23)-C(24)-H(35)	-0.0313	O(15)-C(13)-C(14)-H(31)	-168.1456
C(22)-C(23)-C(24)-C(25)	0.1241	O(12)-B(16)-O(15)-C(13)	53.0326
C(22)-C(23)-C(24)-H(35)	-179.8579	F(17)-B(16)-O(15)-C(13)	-67.9285
C(21)-C(22)-C(23)-O(26)	-179.9137	F(18)-B(16)-O(15)-C(13)	172.1611
C(21)-C(22)-C(23)-C(24)	-0.0884	O(15)-B(16)-O(12)-C(10)	-33.3454
H(34)-C(22)-C(23)-O(26)	0.0667	F(17)-B(16)-O(12)-C(10)	87.8636
H(34)-C(22)-C(23)-C(24)	179.8921	F(18)-B(16)-O(12)-C(10)	-152.4609
C(20)-C(21)-C(22)-C(23)	-0.0191	C(9)-C(13)-O(15)-B(16)	-50.6289
C(20)-C(21)-C(22)-H(34)	-179.9995	C(14)-C(13)-O(15)-B(16)	122.0623
H(33)-C(21)-C(22)-C(23)	179.9922	C(10)-C(9)-C(13)-O(15)	20.7422
H(33)-C(21)-C(22)-H(34)	0.0119	C(10)-C(9)-C(13)-C(14)	-151.7937
C(19)-C(20)-C(21)-C(22)	179.9207	C(8)-C(9)-C(13)-O(15)	-154.3534
C(19)-C(20)-C(21)-H(33)	-0.0912	C(8)-C(9)-C(13)-C(14)	33.1107
C(25)-C(20)-C(21)-C(22)	0.0903	C(5)-C(10)-O(12)-B(16)	-172.4604
C(25)-C(20)-C(21)-H(33)	-179.9216	C(9)-C(10)-O(12)-B(16)	6.4837
C(14)-C(19)-C(20)-C(21)	179.9755	C(8)-O(7)-C(4)-C(3)	-176.3869

Table S4. Calculated TD-DFT singlet vertical excitation wavelengths and corresponding singlet oscillator strengths for complex L1.

λ (nm)		Frontier orbitals involved [a]	f
417.92	$S_0 \rightarrow S_1$	HOMO - - - LUMO (60 %)	0.9204
370.70	$S_0 \rightarrow S_2$	HOMO-2 - - - LUMO (49 %)<br>HOMO-1 - - - LUMO (49 %)	0.0070
360.45	$S_0 \rightarrow S_3$	HOMO-1 - - - LUMO (50 %)<br>HOMO - - - LUMO (13 %)	0.2025

[a] Only the main frontier orbitals involved in the $S_0 \rightarrow S_n$ transitions (> 50 %) are shown.

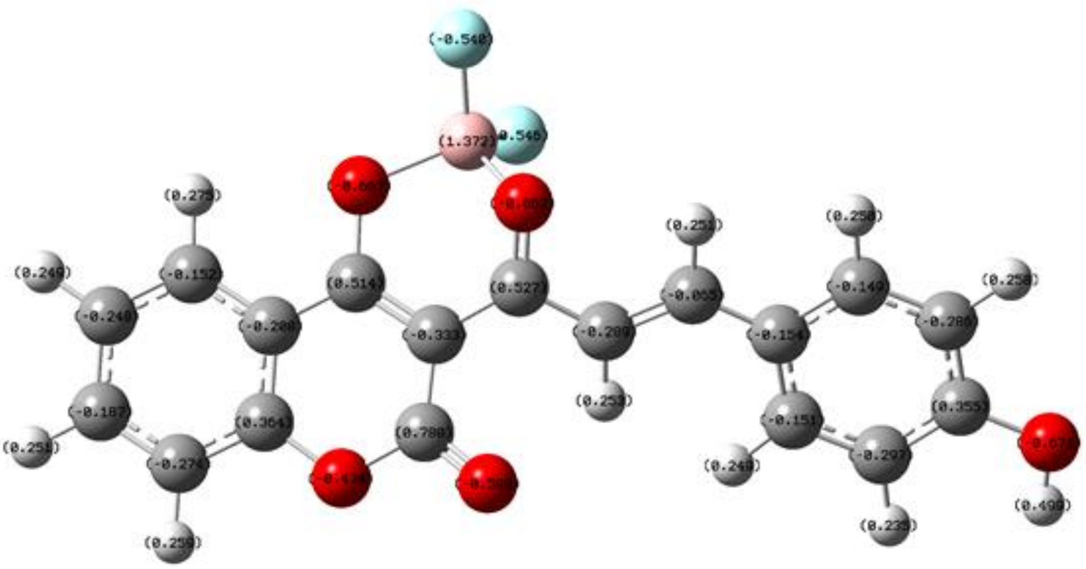

Figure S7. Calculated NBO atomic charges for L1.

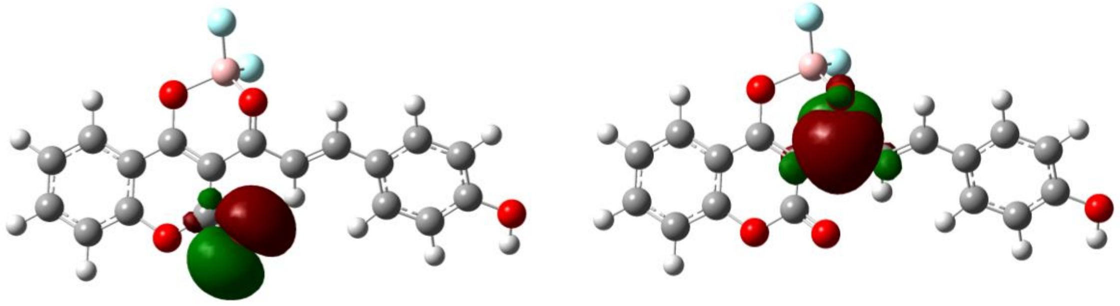

HOMO **LUMO**

Figure S8. Molecular orbital surfaces for the main frontier orbitals (HOMO and LUMO) involved in the S_0 - S_n transitions with the highest singlet oscillator strength.

The geometry of the dimer of the L1 compound has been optimized. As part of the dimer, two molecules are oriented head-to-tail with the formation of intermolecular hydrogen bonds between the hydrogen atom of the hydroxy group of one molecule and the fluorine atom of the other molecule.

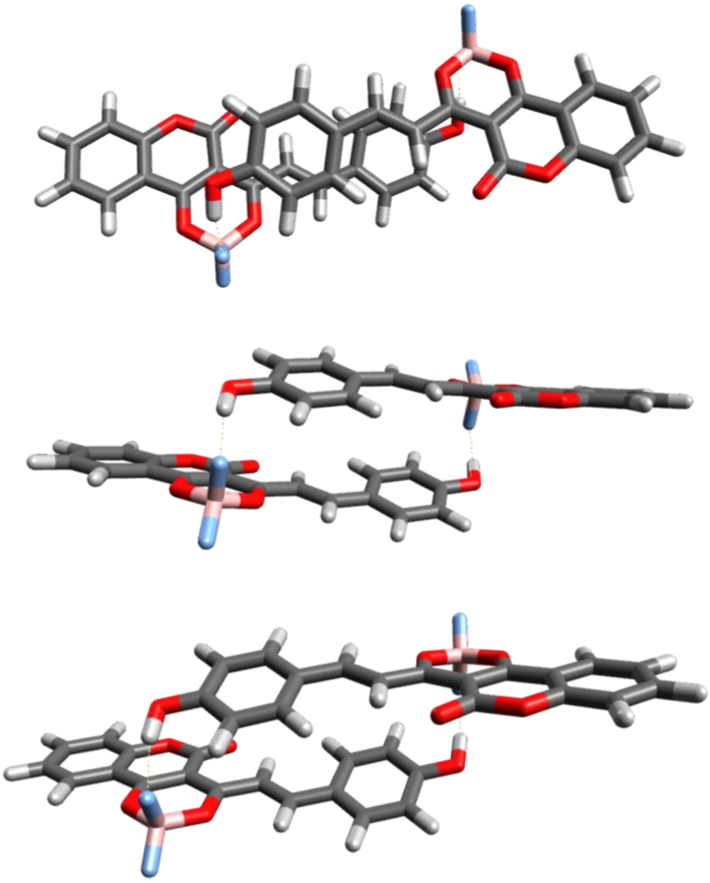

Figure S9. Orientation of the L1 molecules in the dimer.

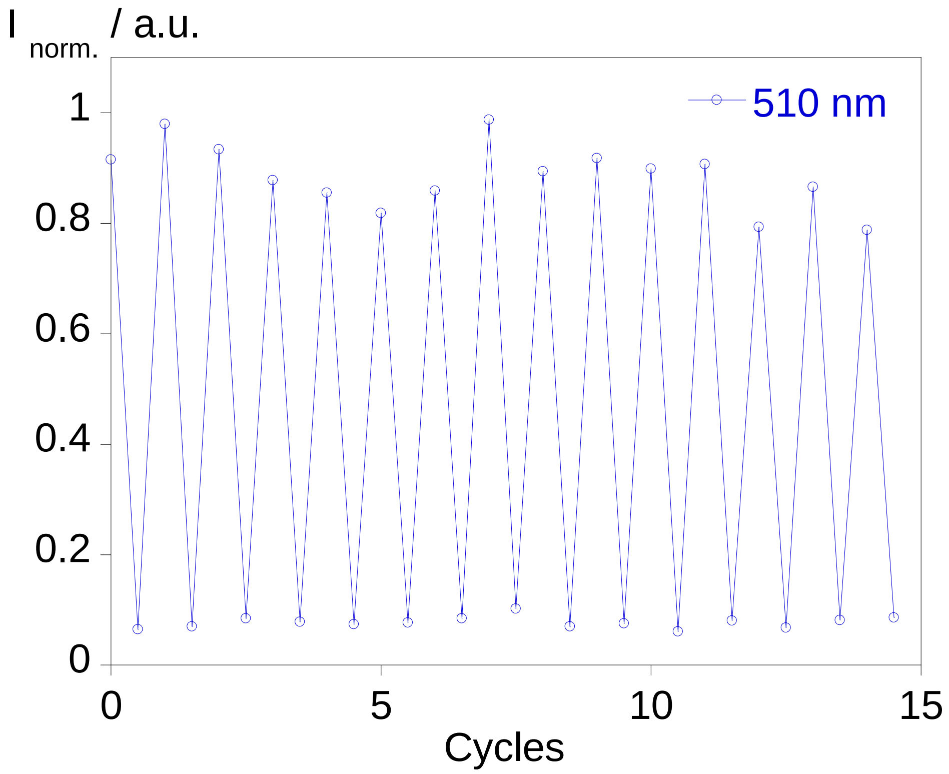

Figure S10. Fluorescence quenching and enhancement cycles with temperature of the L1@PMMA polymer (λ_{em} = 510 nm).

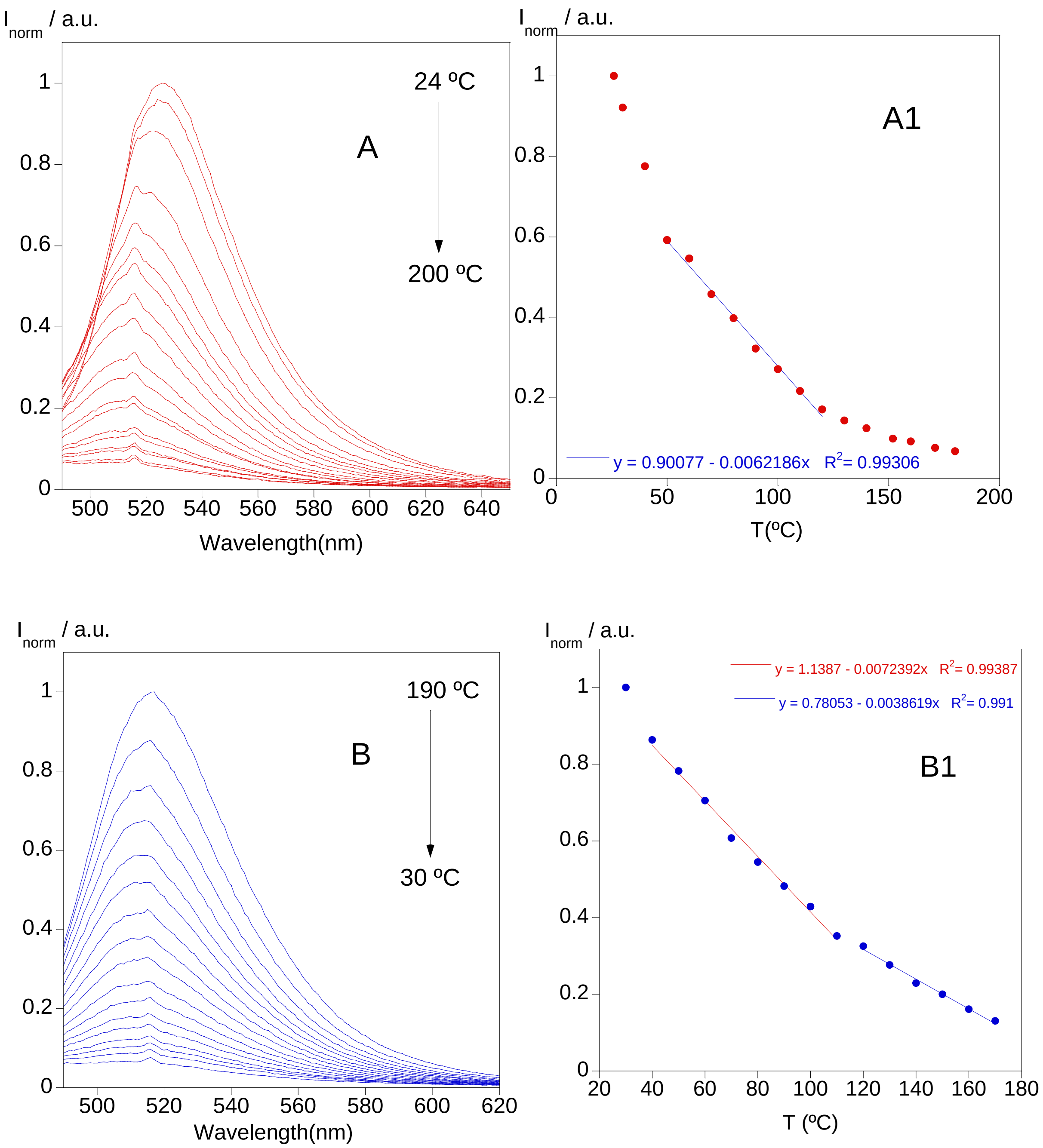

Figure S11. Temperature-dependant emission spectra of L1 in KURARITY™ 4285 A) during heating and B) during cooling, with calibration curves for heating (A1) and cooling (B1) at 525nm.

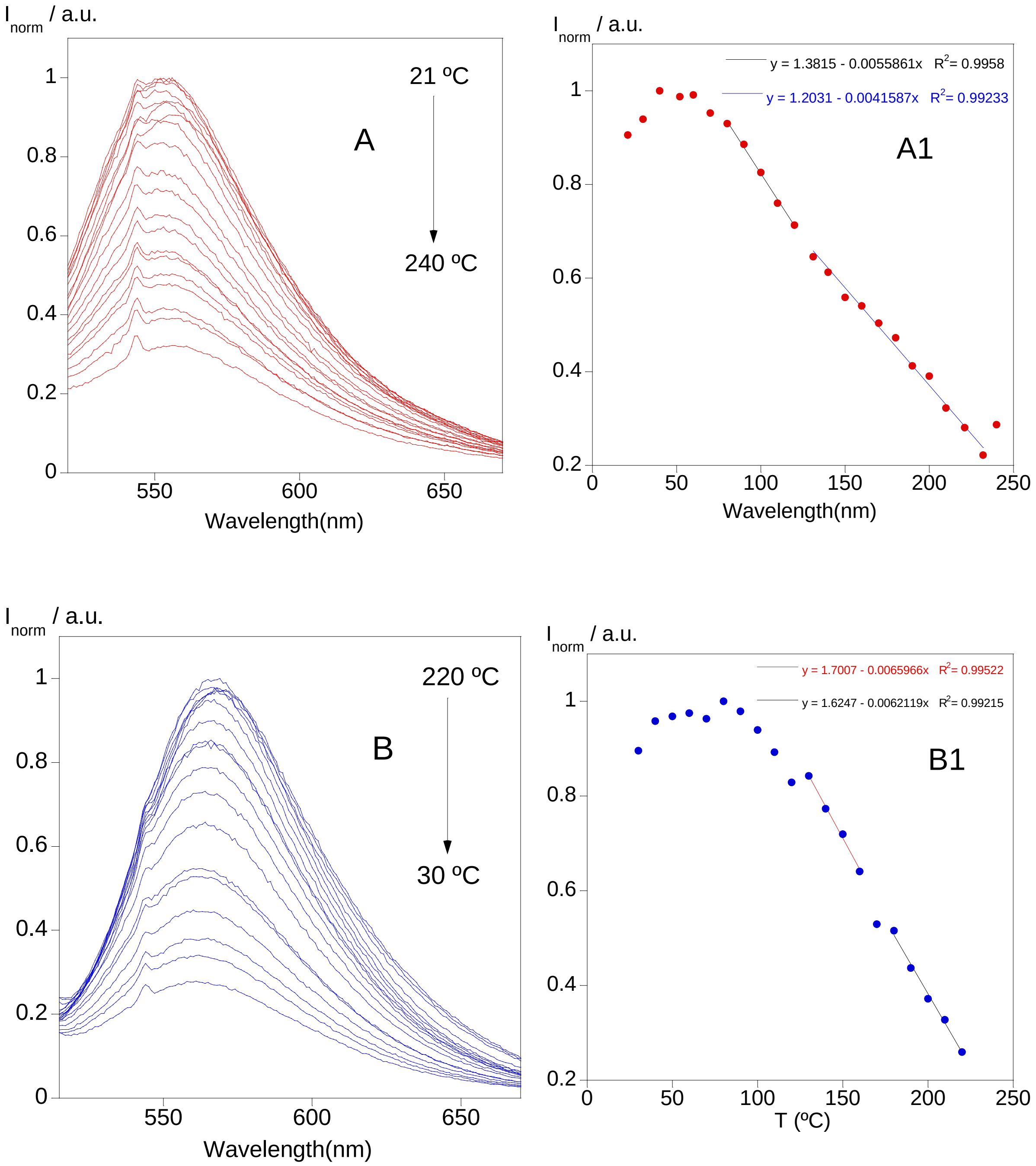

Figure S12. Temperature-dependant emission spectra of L2 in PMMA A) during heating and B) during cooling, with calibration curves for heating (A1) and cooling (B1) at 569nm.

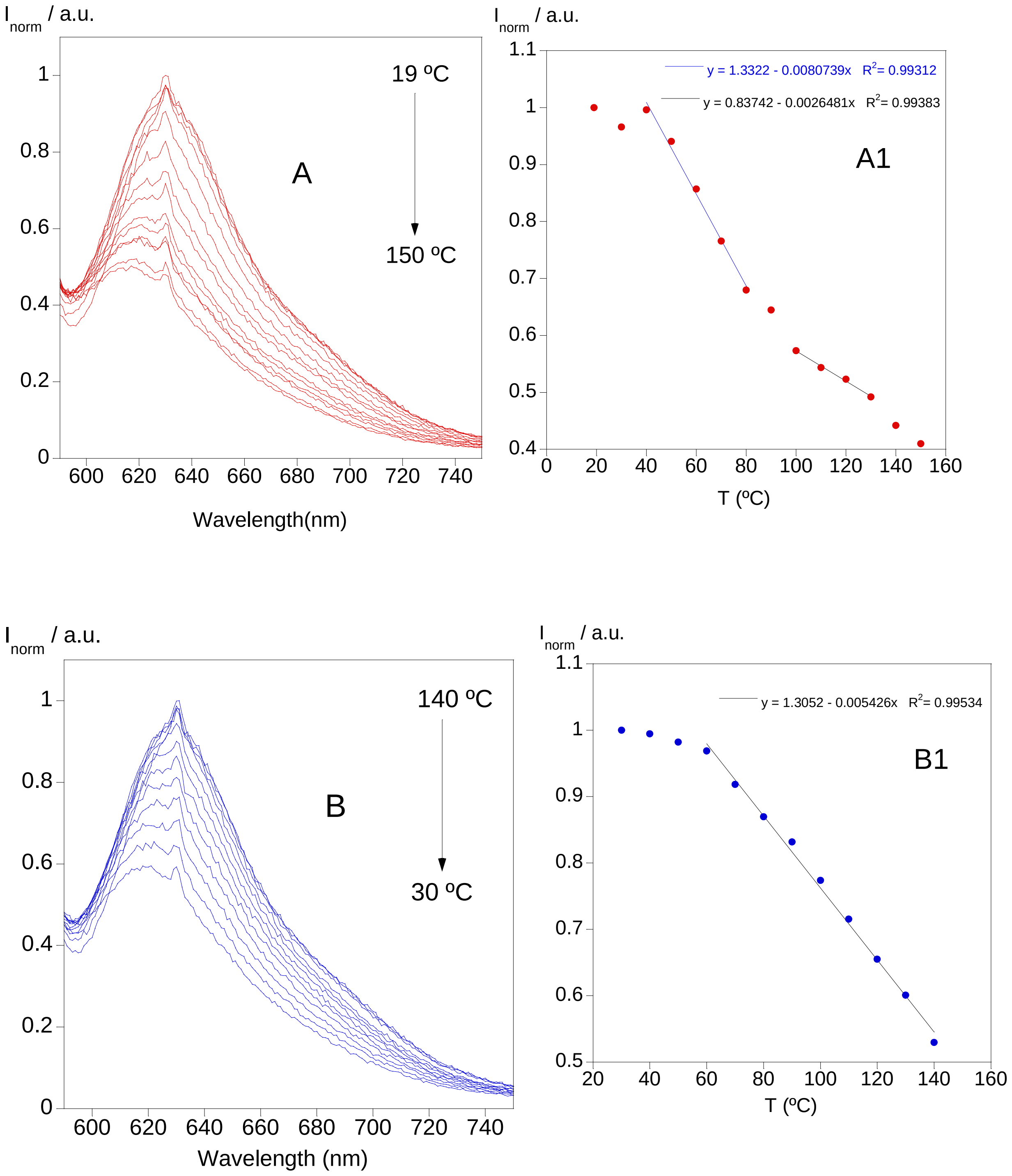

Figure S13. Temperature-dependant emission spectra of L3 in PMMA A) during heating and B) during cooling, with calibration curves for heating (A1) and cooling (B1) at 640nm.

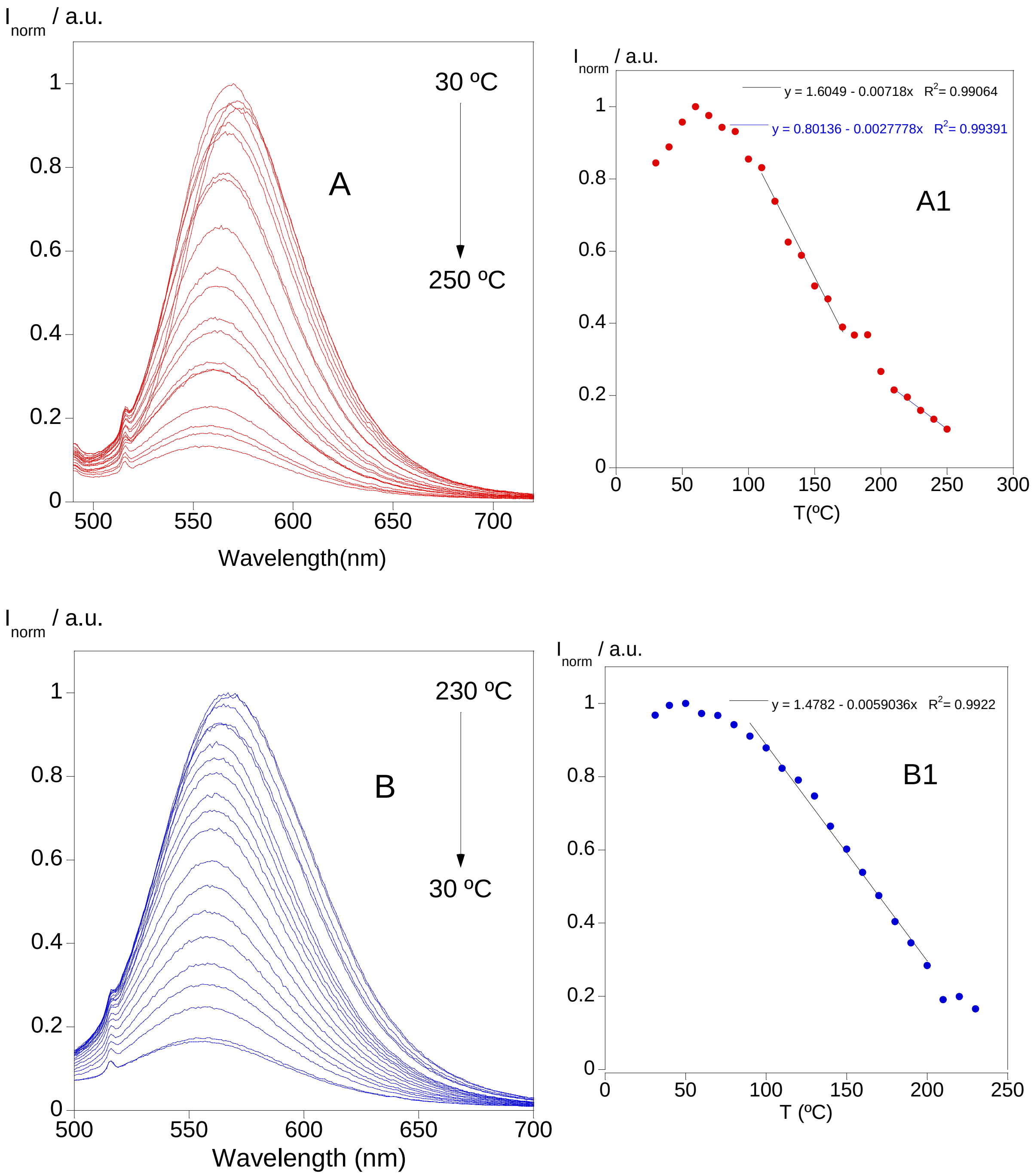

Figure S14. Temperature-dependant emission spectra of L1+L2 in PMMA A) during heating and B) during cooling, with calibration curves for heating (A1) and cooling (B1) at 552nm.

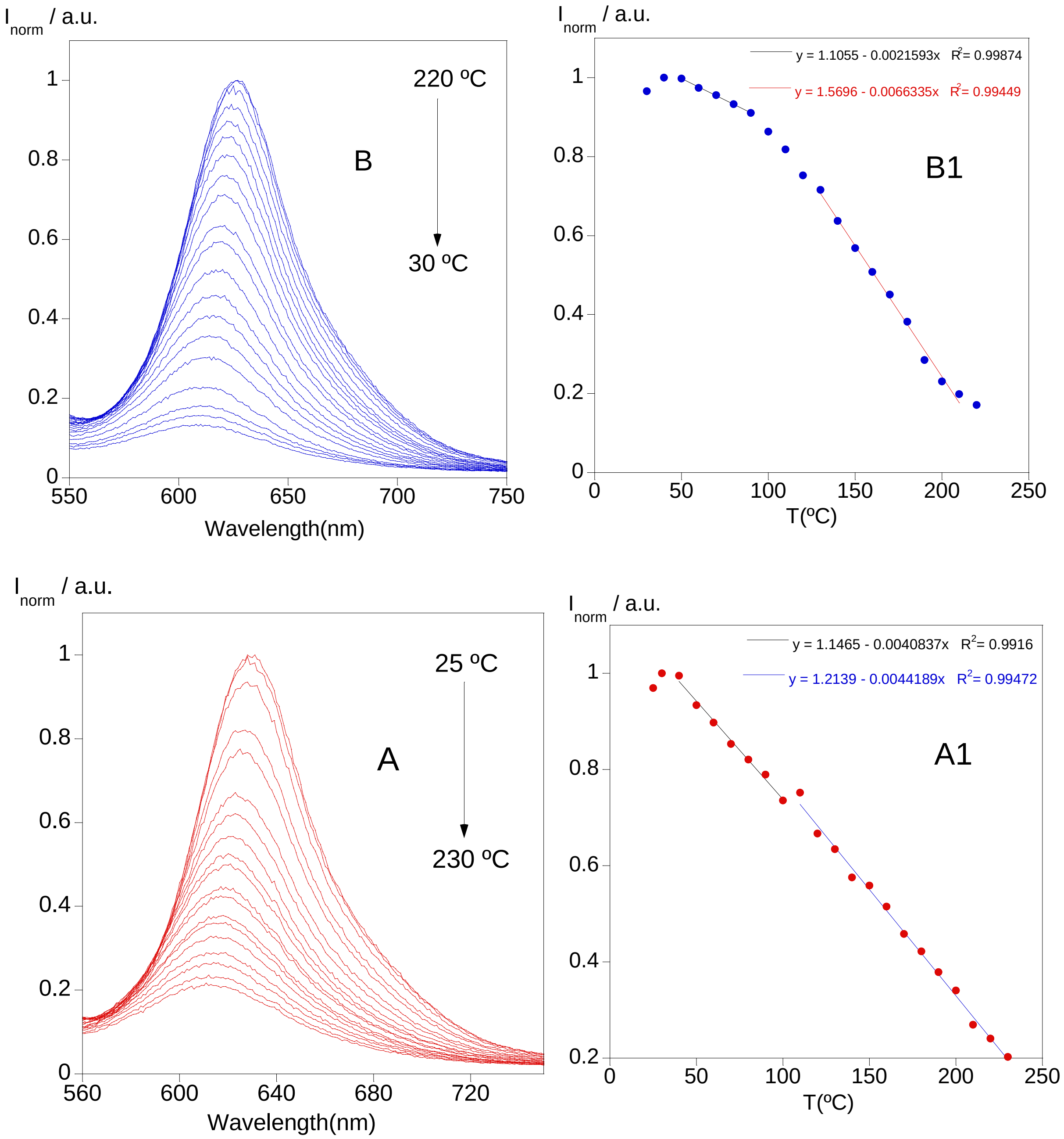

Figure S15. Temperature-dependant emission spectra of L1+L3 in PMMA A) during heating and B) during cooling, with calibration curves for heating (A1) and cooling (B1) at 607nm.

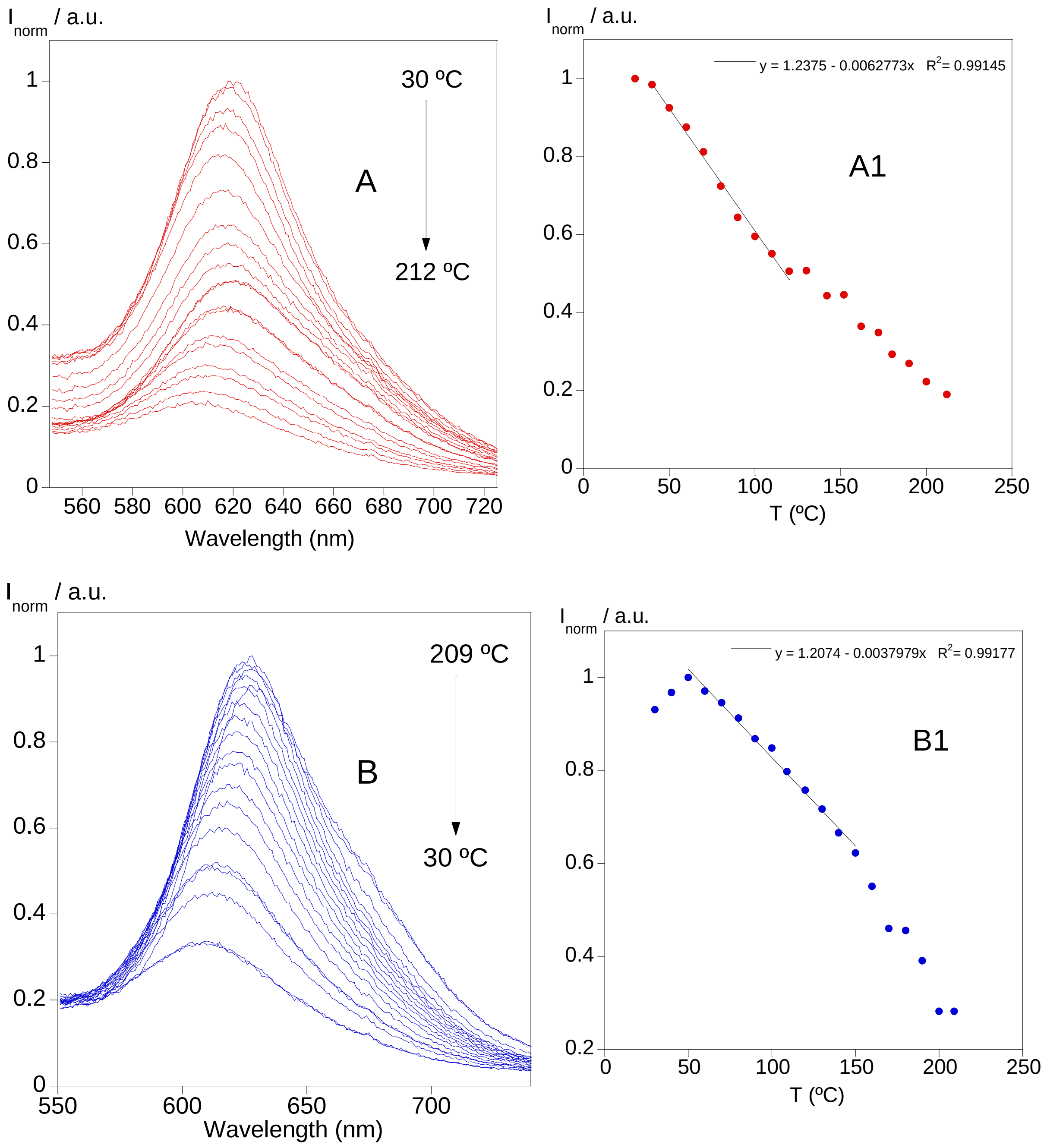

Figure S16. Temperature-dependant emission spectra of L2+L3 in PMMA A) during heating and B) during cooling, with calibration curves for heating (A1) and cooling (B1) at 628nm.

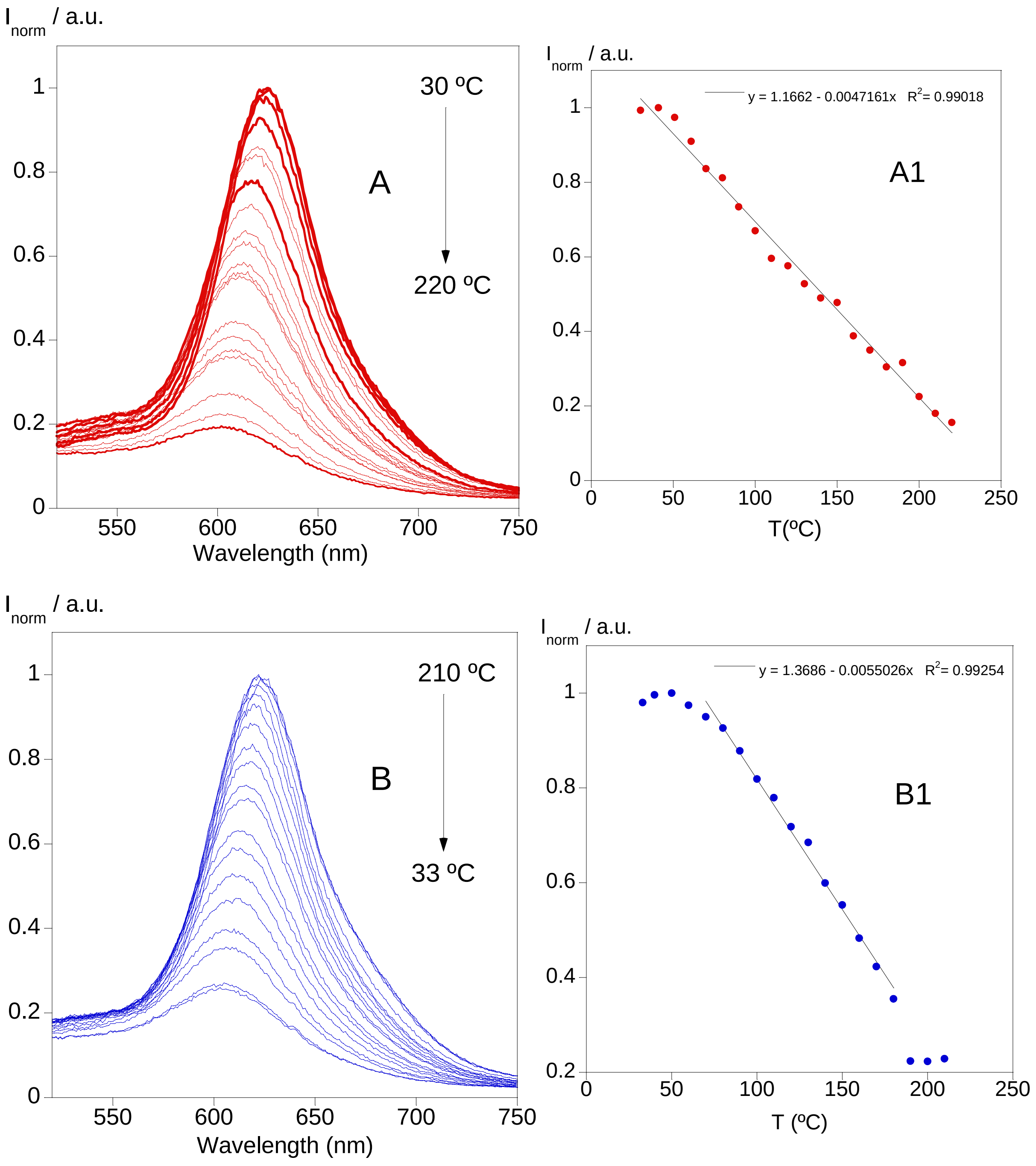

Figure S17. Temperature-dependant emission spectra of L1+L2+L3 in PMMA A) during heating and B) during cooling, with calibration curves for heating (A1) and cooling (B1) at 627nm.

Table S5. Interval(s) of temperature with linear behaviour between TºC and emission intensity of doped PMMA polymer films and percentage of emission recovery after cooling.

Compound(s)	Interval(s) of linearity	Emission recovery after cooling (%)
L1	60ºC -> 120ºC; 180ºC -> 120ºC; 110ºC -> 40ºC	83,7%
L2	80ºC -> 120ºC ; 130ºC ->230ºC; 220ºC -> 180ºC 160ºC-> 130ºC	103,2%
L3	40ºC -> 80ºC; 100ºC -> 130ºC; 140ºC -> 60ºC	100,1%
L1+L2	110ºC -> 170ºC; 210ºC -> 250ºC; 200ºC -> 90ºC	90,1%
L1+L3	40ºC -> 100ºC; 110ºC -> 250ºC; 210ºC -> 140ºC ; 90ºC -> 50ºC	114,9%
L2+L3	40ºC -> 120ºC; 150ºC -> 50ºC	100,2%
L1+L2+L3	30ºC -> 220ºC; 180ºC -> 80ºC	102,7%

Dependence of spectral characteristics on temperature

To assess the effect of rotation of the styrene fragment relative to the coumarin cycle with temperature changes on absorption spectra, the calculation of the energy difference between the HOMO and the LUMO was carried out depending on the angle of rotation relative to one of the single bonds of the styrene fragment in the **L1** molecule.

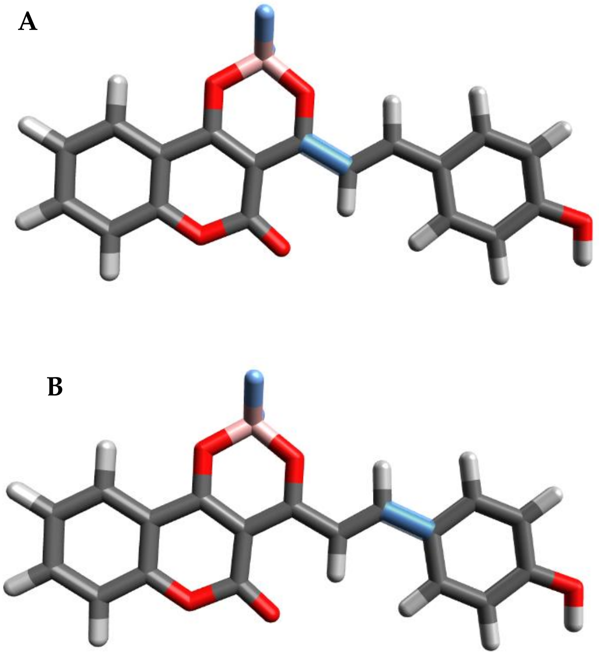

Figure S18. The blue color indicates the bonds with respect to which the dihedral angle changed.

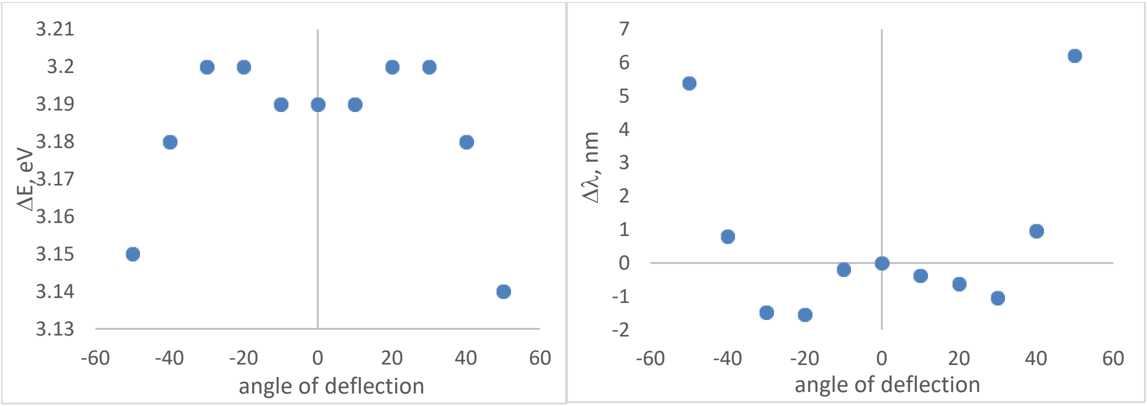

Figure S19. The changes in the energy difference between the HOMO and the LUMO (left) and the position of the absorption maximum (right) with a change in the dihedral angle relative to the bond

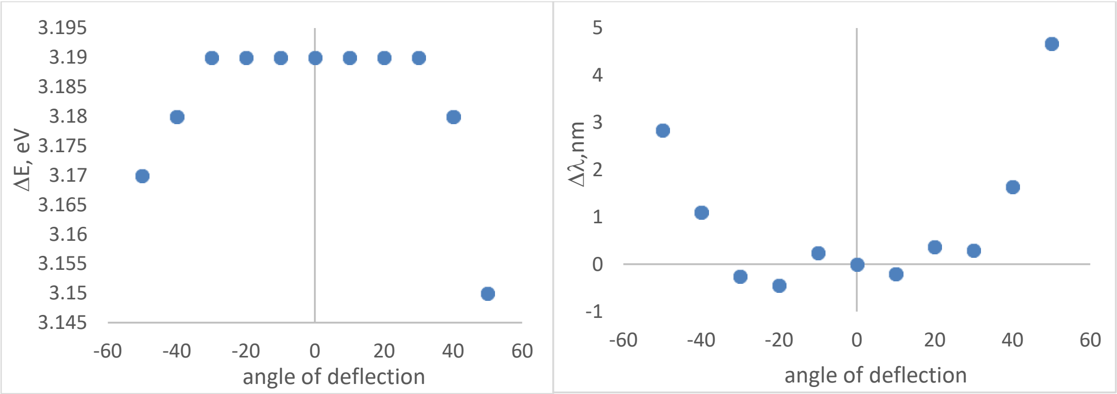

Figure S20. The changes in the energy difference between the HOMO and the LUMO (left) and the position of the absorption maximum (right) with a change in the dihedral angle relative to the bond **B.**

According to the results of calculations, a notable change in the wavelength is expected with a deviation from the planar state of more than 40-50°. Turn around the ordinary bonds even with a deviation of 50°, the expected changes do not exceed 10 nm. It can be assumed that the strength of the oscillator decreases much more significantly with an increase in the dihedral angle.
